# Dynamic Covalent Polymeric Foams: En Route to a Sustainable Lightness

**DOI:** 10.1002/cssc.202502478

**Published:** 2026-03-01

**Authors:** Antoine Adjaoud, Anaë Girault‐Fodil, Farida Baraka, Pierre Verge

**Affiliations:** ^1^ Functional Polymers & Particulate Materials Luxembourg Institute of Science and Technology Esch‐sur‐Alzette Luxembourg; ^2^ Department of Physics and Materials Science University of Luxembourg Esch‐sur‐Alzette Luxembourg; ^3^ Engineering Faculty of Gipuzkoa Chemical and Environmental Engineering Department Biorefinery Processes Group University of the Basque Country Donostia Spain

**Keywords:** dynamic bonds, polymer foams, recycling, sustainability

## Abstract

The broad versatility of polymer foams has driven their industrial development in commodity usages and cutting‐edge technologies seeking lightweight and multifunctionality. The downside of this booming production lies in the limited end‐of‐life options of their waste that poses significant environmental challenges and urges the development of circular alternatives. Building on recent advances in dynamic covalent polymer networks (DCPNs), one promising route lies in the activation or the incorporation of dynamic covalent bonds (DCBs) into polymer foams. Thermally induced topological rearrangements are thus enabled by dynamic exchange reactions, unlocking new functionalities. This concept gave rise to dynamic covalent polymer foams (DCPFs), a class of materials that provide recyclability without compromising the mechanical strength and the dimensional stability traditionally required for cellular materials. In line with the United Nations Sustainable Development Goals (UN SDGs), the emergence of these dynamic networks redefines the value chain of polymeric foams. Strategies for designing DCPFs are truly numerous, all drawing inspiration from the literature on dynamic exchange chemistry. However, a clear review of the strategies, processes, materials, and new functions is still missing. This review tends to fill this gap.

## Graphical abstract

Dynamic covalent polymer foams (DCPFs) combine lightweight cellular structures with adaptive polymer networks, enabling self‐healing, reprocessability, and recyclability. By integrating reversible covalent bonds into foam architectures, these materials open new pathways toward sustainable porous polymers within a circular materials economy.

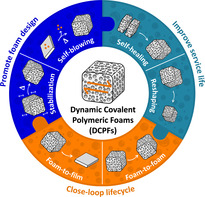

## 1 Introduction

Lightweight, dimensionally stable, and tunable in both design and properties, polymer foams make our life more convenient. From construction sector to packaging, footwear, and automotive industry, polymer foams occupy a strategic position in the plastics market. Emerging applications in filtration, biomedical devices, and healthcare have further broadened the range of foam materials produced [1]. If nearly all cases of plastics can be processed into flexible or rigid foams, their recyclability is strongly governed by their macromolecular structure. Thermoplastic foams, composed of linear polymer chains, can often be recycled after densification via melting or solvent‐based processes, provided the polymer chains remain intact. However, thermoplastic foams are limited by their low thermal resistance, poor dimensional stability under load, and susceptibility to creep, which restrict their use in structural or high‐temperature applications where thermoset foams excel. Conversely, thermoset foams maintain dimensional stability and mechanical integrity under extreme conditions, making them indispensable in high‐performance and safety‐critical sectors. Commercial applications such as insulation and structural materials are largely dominated by polyurethane foams which represent the highest production volumes of polymer foams worldwide. However, their cross‐linked networks hinder melt reprocessing, leading to their disposal and thus contributing to land pollution [2]. Numerous downcycling strategies have been developed to mitigate post‐consumer waste accumulation [3]. For example, mechanical recycling intends to repurpose ground rigid foams as lightweight fillers in construction materials [4]. Chemical recycling pathways such as solvent‐assisted depolymerization has been also widely explored [5, 6]. Although these multistep approaches show promise at the laboratory scale, they remain economically and energetically demanding for industrial implementation, often requiring harsh solvents, expensive catalysts, extreme temperature, or pressure conditions to recover heterogeneous and low‐purity recycled products. There is a clear need for viable alternatives that can genuinely enhance end‐of‐life management while preserving the advanced functional properties of these materials. A promising strategy lies in the field of dynamic covalent polymers which rely on reversible bonds that enable network rearrangement and confer unique recyclability aptitudes to otherwise permanently cross‐linked materials.

**FIGURE 1:**
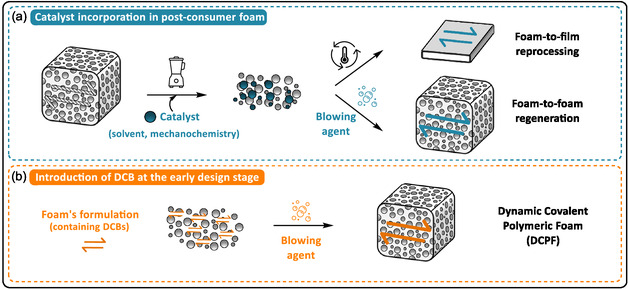
Strategies developed to design dynamic covalent polymer foams (DCPFs): (a) catalyst incorporation in post‐consumer foam to activate dormant DCB and (b) introduction of DCB at the early design stage.

Dynamic covalent polymers are a class of polymers whose network rearrangement relies on reversible bond exchange reactions occurring under dynamic equilibrium. The concept of dynamic equilibrium in polymer chemistry was initially explored through supramolecular assemblies based on noncovalent interactions [7]. Extending this concept to covalent systems has given rise to dynamic covalent polymer networks (DCPNs), also referred to as covalent adaptable networks (CANs), which incorporate dynamic covalent bonds (DCBs) in their cross‐linked networks [8, 9]. Unlike permanent covalent bonds, DCBs can reversibly break and reform under external stimuli such as heat, light, or pH. This reversibility facilitates topological rearrangements, giving cross‐linked polymers the ability to be reshaped, self‐healed, and reprocessed [10]. When transposed to foams, integrating DCBs offers new opportunities for reuse and recycling. In this framework, the design of dynamic covalent polymeric foams (DCPFs) follows two main strategies: (1) the activation of latent dynamic bonds via catalyst incorporation and (2) the introduction of DCBs during the early‐stage of foam precursors design (Figure 1). Dynamic exchange reactions not only extend material lifetimes. They also enable innovative features at various stages of their lifecycle.

After a general introduction, this review is structured into three main sections. The first section provides an overview of the dynamic covalent chemistries that have been implemented in polymer foams, highlighting their underlying mechanisms and material design considerations. The second section examines how these chemistries translate into enhanced functionalities in cellular materials, including adaptability, self‐healing, reprocessability, and recyclability. Finally, the review concludes with an objective SWOT (strengths, weaknesses, opportunities, and threats) analysis to critically assess the potential of dynamic covalent polymer foams in the context of sustainability and alignment with the United Nations Sustainable Development Goals (UN SDGs).

## 2 Generalities

### 2.1 Polymer Foams

**SCHEME 1:**
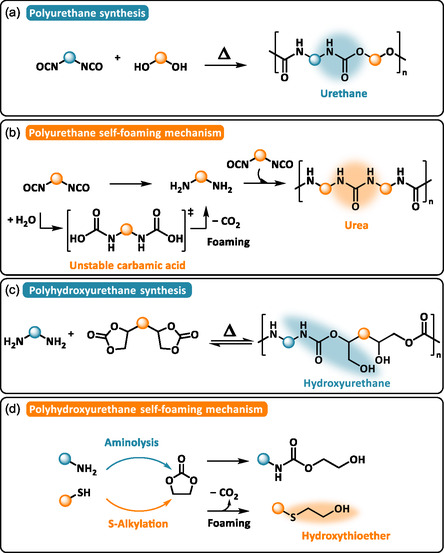
Synthetic approaches for (a) polyurethanes and (c) polyhydroxyurethanes and their respective self‐foaming mechanisms through decarboxylative (b) hydrolysis of isocyanate and (d) S‐alkylation of five‐membered cyclic carbonate.

Foaming of synthetic polymers relies on precise control of processing parameters and functional precursors. Thermoplastic foams are obtained from preformed polymers in the molten state via extrusion or injection foaming, while thermosetting foams are produced during simultaneous cross‐linking and gas generation. In both cases, a blowing agent is required to initiate cell nucleation and generate gas evolution before the foam structure is stabilized [1]. The nature of the blowing agent, either physical or chemical, determines the final foam morphology. Physical blowing agents include compressed gases such as nitrogen (N_2_) or supercritical carbon dioxide (Sc‐CO_2_), and volatile solvents that evaporate or sublimate when heated or cooled. These external agents physically create the cellular structure [1]. Chemical blowing agents are compounds that instead decompose upon thermal activation to release gases in a controlled manner, allowing precise tuning of cell nucleation and growth. These agents are usually added to the matrix and react within a specific viscosity and temperature range. Alternatively, the blowing agent can be embedded directly into the molecular structure of polymer precursors, creating the concept of self‐foaming polymers as defined by the group of Detrembleur [11]. Self‐foaming polymers autonomously form and stabilize a cellular structure during polymerization without the addition of external blowing agents. While self‐foaming polymers often rely on self‐blowing reactions in which the blowing gas is generated in situ through chemical reactions, gas generation alone does not necessarily guarantee stable foam formation. Polyurethanes (PUs) are a leading example of self‐foaming polymers. They are formed by reacting polyols and isocyanates (Scheme 1a), and partial hydrolysis of isocyanate groups generates CO_2_ which acts as internal blowing agent (Scheme 1b). Compared to conventional foaming approaches, self‐foaming strategies simplified formulations by reducing reliance on volatile or potentially hazardous additives. The intrinsic harmonization between self‐foaming and crosslinking reactions can enhance cell stability, homogeneity of the cellular morphologies, and process robustness.

However, the toxicity of isocyanates has driven the development of safer non‐isocyanate polyurethane (NIPU) alternatives [12]. Among them, poly(hydroxyurethane)s (PHUs) synthesized from the ring‐opening of cyclic carbonates with polyamines have attracted interest (Scheme 1c), even though their self‐foaming typically requires specific reaction conditions [13]. To overcome this limitation, Detrembleur et al. proposed a dual chemo‐ and regio‐selective ring‐opening strategy of cyclic carbonate using amines and thiols [14]. In this method, the S‐alkylation of cyclic carbonate forms hydroxythioether linkages and releases CO_2_ internally, enabling the synthesis of self‐foaming non‐isocyanate polyurethane foams (NIPUFs) (Scheme 1d). Today, a variety of design strategies have been developed to tailor foams properties, improve compatibility with industrial processing methods, and close the loop of their lifecycle.

### 2.2 Dynamic Covalent Polymer Networks (DCPNs)

**FIGURE 2:**
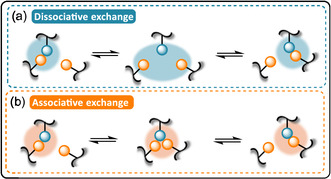
General representation of (a) dissociative and (b) associative dynamic exchange reaction in dynamic covalent polymer networks (DCPNs). Modified with permission [15]. Copyright 2019, American Chemical Society.

DCPNs are cross‐linked polymers that contain reversible bonds allowing for topological rearrangements when exposed to external stimuli. These exchange reactions facilitate mechanical stress relaxation and endow materials, typically incapable of such behaviors, with reshuffling and recycling capabilities. Early examples of DCPNs relied on dissociative exchange mechanisms. In these systems, bond dissociation precedes bond reformation (Figure 2a). The reversible Diels–Alder (DA) reaction between furan and maleimide is a very popular example of dissociative exchange [15, 16]. At high temperature, dissociative DCPNs depolymerize and tend to exhibit a thermoplastic‐like flow behavior. This abrupt loss of dimensional stability limits the use of these materials to a specific range of temperature, typically below 90°C for DCPNs relying on DA to avoid retro‐Diels Alder (rDA) reaction. To tackle this issue, associative dynamic chemistries were introduced by Leibler and coworkers in cross‐linked network (Figure 2b) [17, 18]. In this subclass of DCPNs (also known as vitrimers), bond formation and dissociation occur together, maintaining a permanent network connectivity even at elevated temperatures. In these systems, the network cross‐link density and dimensional stability are preserved over a wider range of temperature. In both types of reversible networks, temperature‐induced dynamic exchange reactions enable stress relaxation through topological rearrangements, imparting the advantageous self‐healing, reshaping, and reprocessing properties characteristic of DCPNs [19].

Initially, vitrimers were developed by incorporating zinc‐based catalysts into epoxy–anhydride formulations, allowing for dynamic exchange reactions between ester and hydroxyl groups [17]. Catalysts lower the activation energy of bond exchange, by interacting with the functional groups responsible for the dynamic exchanges [20]. However, they are not covalently bound to the network, and may leach out from the network, leading to human and environmental impacts. The activation of dynamic exchange reactions can also be triggered with internal catalyts, which are inherently connected to the molecular structure of the network [21, 22]. Various dynamic chemistries and their derivatives have been explored to date and standardized experimental workflows have been established to assess their dynamic behavior [15, 23]. All these strategies received considerable attention toward renewable development [24, 25] and industrial implementation [26], highlighting their strong potential in circular polymer manufacturing.

## 3 Application of Dynamic Covalent Chemistry to Polymer Foams

### 3.1 Catalytic Activation Strategies for Post‐Consumer Foams

**SCHEME 2:**
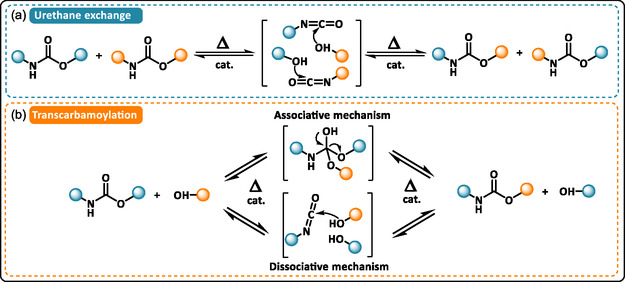
General reaction of mechanism of: (a) urethane exchange via dissociative mechanism and (b) transcarbamoylation via both associative and dissociative mechanism. Modified with permission [27]. Copyright 2022, American Chemical Society.

Polyurethane foams (PUFs) are the most widely used polymeric foams, yet the dynamic nature of urethane bonds was long overlooked. Studies have shown that urethane bonds can reversibly exchange through two mechanisms: urethane–urethane exchange (dissociative, Scheme 2a) and transcarbamoylation between urethane and hydroxyl groups, which can either be associative or dissociative depending on the catalyst (Scheme 2b) [27]. This dynamic behavior, first reported by Offenbach and Tobolsky [28], was later revived by Xie and coworkers who observed stress relaxation in thermoset PUs containing dibutyltin dilaurate (DBTDL) catalyst [29]. Their results suggested that, in addition to promoting PU formation, DBTDL also accelerates reversible exchange reactions at elevated temperatures. Before the emergence of DCPNs, Quadrini et al. demonstrated that commercial thermoset PUFs could be reprocessed into films by compression molding, likely due to the presence of such exchangeable urethane bonds and catalysts [30]. Caillol and coworkers further confirmed temperature‐dependent stress relaxation in the range of 160°C–200°C in DBTDL‐containing PUFs, most probably induced by dissociative transcarbamoylation [31]. Later, Dichtel et al. showed that 1,4‐diazabicyclo[2.2.2]octane (DABCO) gelling agent and an excess of hydroxyl groups favored an associative mechanism [32]. Collectively, these studies showed that the introduction of catalysts during PUF design enables the reprocessing of crosslinked PU thanks to the activation of urethane‐based reversible exchange [31, 32, 33, 34]. Bottom‐up and post‐synthetic strategies have been explored to introduce catalyst into a cured network containing dormant dynamic bonds and promote conversion into a dynamic network (Figure 1a) [35]. Building on this, Dichtel and co‐workers introduced urethane‐exchange catalysts into post‐consumer PUFs via solvent immersion, allowing foam scraps to be reprocessed into films with glass transition temperatures similar to the original material (*T*_g_ = 52°C and 53°C, respectively) [36]. They later replaced tin‐based catalysts with less toxic zirconium analogs, enabling multiple reprocessing cycles while preserving mechanical integrity (*σ*_break_ = 30 and 29 MPa for the first and the fifth reprocessed films, respectively) [37]. Mechanochemical methods have been also investigated to uniformly disperse catalyst into ground PUFs [38, 39]. Luo and coworkers showed that ball milling improves catalyst distribution and surface activation [38]. Using *para*‐Toluenesulfonic acid (*p*TSA) and glycerol, they promoted associative transcarbamoylation without inducing depolymerization [39]. However, other studies showed that excessive catalyst loading can lead to depolymerization through dissociative‐based mechanisms. Bandegi et al. introduced triazabicyclodecene (TBD) as an organocatalyst into commercial rigid PUF [40]. Increasing the catalyst concentration from 2 to 10  wt% led to a reduced storage modulus in the rubbery state and an abrupt modulus drop around 170°C, consistent with dissociative urethane exchange and depolymerization.

Overall, introducing selective catalysts into pre‐existing foams containing latent dynamic bonds represents a cost‐effective strategy for recycling and upcycling PUFs (Table 1). This approach is compatible with industrial processes such as compression molding, microcompounding, and foam injection molding [41] and could be extended to other chemistries with built‐in exchangeable linkages, supporting material circularity, and extended product lifetimes.

**TABLE 1:** Selected examples of catalyst introduction in post‐consumer foams.

Polymer foam (supplier or composition)	Catalyst (concentration)	Mixing method (conditions)	Reprocessing method (conditions)	Properties of reprocessed films	References
Commercial PUF (Airlite)	DBTDL^a^ (0.64 wt%)	Solvent‐assisted (30 mg/mL in CH_2_Cl_2_,overnight)	Twin‐screw extrusion (*ω *= 100 rpm, *T* = 220°C, *t* = 1 min)	τ*_160°C_ = 40 s^b^ *T*_*α*_ = −45°C^c^ *E* = 2.9 GPa^d^	[36]
Model PUF (polyester polyol^e^, MDI^f^)	Zr(acac)_4_ g (3 wt%)	Cryogenic milling (*ω *= 1800 rpm, *t* = 20 min)	Twin‐screw extrusion (*ω *= 50 rpm, *T* = 200°C, *t* = 1 min)	τ*_160°C_ = 54 s *T*_g_ = 32°C^h^ *E*′ = 28 MPa^i^	[37]
Commercial PUF (Hongshun composites)	DBTDL (1 wt%)	Ball milling (*ω *= 600 rpm, *t* = 60 min)	Compression molding (*P* = 30 MPa, *T* = 170°C, *t* = 30 min)	τ*_170°C_ = 196 s *T*_g_ = 102°C *σ *= 33 MPa^j^	[38]
Commercial PUF (Hongshun composites)	*p*TSA^k^ & GLY^l^ (5 & 1 wt%)	Ball milling (*ω *= 600 rpm, *t* = 90 min)	Compression molding (*P* = 30 MPa, *T* = 130°C, *t* = 45 min)	τ*_130°C_ = 820 s *T*_g_ = 119°C *σ *= 27 MPa	[39]

a Dibutyltin dilaurate.

b Stress relaxation time.

c α‐Relaxation temperature corresponding to maximum of tan δ curve (DMTA).

d Young modulus determined by strain‐stress experiment.

e Poly[trimethylolpropane/di‐(propylene glycol)‐alt‐adipic acid/phthalic anhydride] polyol.

f Methylene diphenyl diisocyanate.

g Zirconium acetylacetonate.

h Glass transition temperature.

i Storage modulus at 110°C determined by DMTA experiment.

j Tensile strength.

k *para*‐Toluenesulfonic acid.

l Glycerol.

### 3.2 Early‐Stage Integration of Dynamic Covalent Bonds

Recent research on polymer foams have focused on implementing innovative DCBs during the material design stage. Incorporating reversible linkages that are not typically found in commercial polymer foams offers a promising strategy to confer unique recycling capabilities and fine‐tune the temperature range at which network rearrangements occur. However, these DCBs must be carefully selected to avoid disrupting foam formation, particularly gas diffusion and coalescence. At the same time, they must remain active throughout the foam's lifetime to provide dynamic functionality while maintaining dimensional stability in the temperature range of the reversible exchange. The following sections present chemistry‐based strategies for implementing various types of dissociative and associative reversible bonds into the design of dynamic covalent polymer foams (DCPFs).

#### 3.2.1 Diels–Alder Chemistry‐Based DCPFs

**SCHEME 3:**
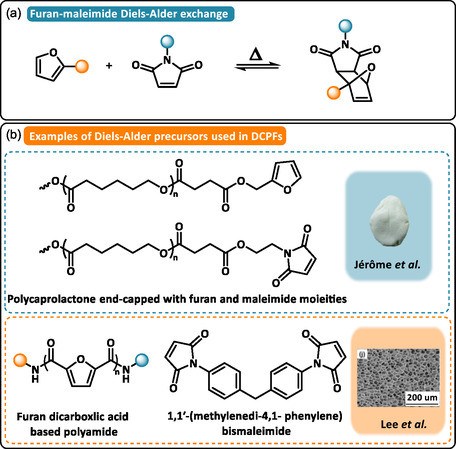
(a) General reaction mechanism of furan‐maleimide Diels–Alder exchange. (b) Examples of Diels–Alder precursors used in DCPFs: Polycaprolactone end‐capped with furan and maleimide moieties. Reproduced with permission [43]. Copyright 2022, Royal Society of Chemistry. Furan dicarboxylic acid based polyamide and 1,1′‐(methylenedi‐4,1‐phenylene) bismaleimide. Reproduced with permission [44]. Copyright 2025, Elsevier.

Diels–Alder (DA) reactions, which occur between electron‐rich dienes (e.g. furan) and electron‐deficient dienophiles (e.g. maleimide), have been studied for almost a century to develop self‐healable materials (Scheme 3a) [16]. DA adducts are formed by [4 + 2] cycloaddition under relatively mild conditions and high selectivity (*T* ≈  60°C). The reverse reaction, i.e. retro‐DA (rDA) reaction, occurs at higher temperature and regenerates starting materials (*T* > 90°C). In the temperature range of rDA, dissociation contributes to the reduction of the cross‐linking density and leads to partial deconstruction of the network and loss of the dimensional stability.

In polymer foams, Aubert and coworkers take advantage of rDA to facilitate the dismantlement of electronic components [42]. Foams were prepared in a two‐step approach, by first reacting maleimide end‐capped siloxane derivatives with furfuryl glycidyl ether, in which succinimide DA‐dynamic crosslinks were formed at 60°C. In a second step, epoxides were reacted with cycloaliphatic amines to form permanent crosslinks. Resulting foams were depolymerized and solubilized in butanol at 90°C as rDA was triggered at this temperature. The thermoplastic‐like flow behavior of materials containing DA bonds was exploited by Houbben et al. to control the foamability and the recyclability of PCL‐based polymers [43]. The foams were synthesized by coupling low‐molecular‐weight PCL stars end‐capped either with furan or maleimide groups (Scheme 3b). Foams were obtained under Sc‐CO_2_ conditions and by heating the material in the temperature range of DA/rDA reactions (*T* = 60°C–90°C). Low‐density foams (*ρ *≈  20 kg·m^−3^) with closed‐cell morphology were successfully obtained. A similar strategy was followed by Lee and coworkers to design reprocessable and refoamable DCPFs [44]. The dynamic network was synthesized from the DA‐cycloaddition between 1,1′‐(methylenedi‐4,1‐phenylene) bismaleimide (BMI) dienophile and a bio‐based diene obtained from the copolymerization of 2,5‐furandicarboxylic acid (FDCA), 11‐aminoundecanoic acid, and Priamine 1075 dimer diamine (Scheme 3b). In comparison to the non‐dynamic formulation, the BMI dynamic crosslinker provided superior melt strength, improving the Sc‐CO_2_ foaming performance. In the temperature range of DA reactions (*T* = 60°C–90°C), the expansion ratio (Ф) gradually increased from 3.1 to 16.9. Finally, the DA crosslinked DCPFs proved to be easy to recycle and remanufacture into new foams with similar elongation and expansion ratio after five refoaming.

In summary, DA‐based dynamic chemistry represents a versatile strategy for engineering sustainable polymer foams. While the low–viscosity mixture obtained above the temperature for rDA reaction can facilitate recycling, external factors can impede DA bonds reformation as a result of structural changes. These changes directly affect the physical and mechanical properties of the foam, even degrading its dimensional stability. Kinyanjui and Hatchett comprehensively documented these effects of thermal exposure of epoxy foams within the temperature range of DA and rDA reactions [45]. The structural changes contributed to irreversible thermal expansion, thus lowering the density and the structural rigidity of the foam. To preserve dimensional stability over a broader temperature range, most DCPFs are therefore designed with associative DCBs, or with DCBs capable of both associative and dissociative behavior.

#### 3.2.2 Urethane‐Based DCPFs

**SCHEME 4:**
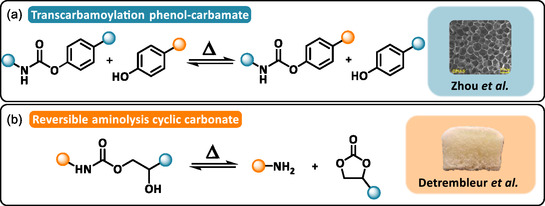
General reaction mechanism of: (a) phenol‐carbamate transcarbamoylation. Reproduced with permission [49]. Copyright 2024, Elsevier. (b) Reversible aminolysis of cyclic carbonate. Reproduced with permission [52]. Copyright 2022, American Chemical Society.

Controlling the dynamic behavior of urethane bonds appears to be a major challenge for the large‐scale recycling of PUFs. Urethane exchange and transcarbamoylation reactions occur slowly at typical reprocessing temperature (120°C–170°C), so most PUs require selective catalysts to accelerate network rearrangements within practical timescales [31, 32]. However, external catalysts can lead to issues such as depolymerization and catalyst migration, which progressively deteriorate performance after repeated recycling. To address these limitations, Xie and coworkers have developed a catalyst‐free strategy based on reversible aromatic urethane linkages [46]. Switching the aromaticity to the oxygen side, Xu et al. showed that using phenol derivatives as polyols and adjusting the electronic properties of the benzene substituents contribute to reduce the dissociation temperature of phenol–carbamate bonds as low as 70°C (Scheme 4a) [47]. Inspired by this concept, Zhou and coworkers developed a series of PUFs containing dynamic phenol–carbamate bonds [48, 49, 50]. Polyphenols were synthesized through the Friedel–Crafts alkylation of vegetable oils (tung oil or cardanol) with *para*‐unsubstituted phenols (guaiacol or catechol). The resulting PUFs were prepared by mixing the polyphenol with a polyisocyanate and cyclopentane as the blowing agent. Curing and foaming were performed at 80°C for several hours, leading to low‐density foams with closed‐cell morphologies, tunable compressive strength ranging from 0.2 to 0.6 MPa. They can also be reprocessed multiple times into films that recover 99% of their tensile strength [49].

The growing demand for renewable and recyclable cellular materials has increased interest in non‐isocyanate polyurethanes (NIPUs) as sustainable alternatives to conventional PUFs [12]. Among them, polyhydroxyurethane (PHU) networks are particularly attractive because of their intrinsic dynamic behavior, which occurs either through associative transcarbamoylation exchange or dissociative cyclic carbonate aminolysis (Scheme 4b) [51]. Self‐foaming PHU obtained via the decarboxylative S‐alkylation of cyclic carbonates have shown strong recyclability potential [52, 53, 54, 55, 56]. Monie and coworkers were the first to show that self‐foaming NIPUFs could be upcycled into new materials [52]. In their system, masked thiols (thiolactones) released CO_2_ during a domino reaction involving amines and cyclic carbonates, imparting latent self‐foaming capability. In the absence of any catalyst, NIPUFs were produced via a free‐rise method to generate soft and rigid foams with open‐cell morphology and densities as low as 167 kg·m^−3^. Using a similar trifunctional cyclic carbonate precursor, Torkelson and coworkers adopted a rheology‐guided strategy to decouple gelation and foaming in self‐blown NIPUFs [53]. By precisely controlling the gelation kinetics, they optimized the foaming window and achieved well‐defined foam morphologies within 30 min at 120°C. In subsequent studies, this approach was extended using cyclic carbonates derived from cardanol [54] or dimer‐acid [55], enabling tunable foams architecture. A systematic design‐of‐experiments approach further correlated the concentration and functionality of thiol‐based precursors with foam morphology and mechanical properties. In all cases, the intrinsic dynamic chemistry of PHUs enabled foam‐to‐film reprocessing with superior recycling efficiency, and full recovery of crosslink density and material performance after multiple recycling (*E*′_80°C_ = 3.63, 3.66, and 3.64 MPa for the first, second, and third remold films, respectively) [53].

Multiple strategies have been developed to simplify PU formulations and design PUFs with controlled dynamic behavior. Since undesired dissociative mechanisms can compromise both mechanical integrity and recyclability [40], catalyst‐free systems have emerged as particularly promising routes toward recyclable PUFs. To further adjust the reworking temperature and enhance recyclability, recent studies have explored dual‐dynamic strategies by introducing additional dynamic bonds not naturally present in PU networks, such as reversible ester [55, 57], disulfide [58, 59], or imine bonds [60]. These examples of dual‐dynamic PUF networks will be discussed in the following sections.

#### 3.2.3 Ester‐Based DCPFs

**SCHEME 5:**
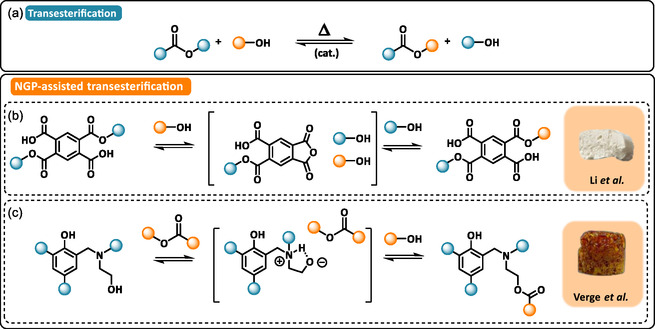
(a) General reaction mechanism of transesterification. Examples of neighboring group participation involved in self‐catalyzed transesterification‐based DCPFs: (b) transesterification via intermediate anhydride in PET foam. Reproduced with permission [62]. Copyright 2022, Royal Society of Chemistry. (c) Transesterification via intermediate 5‐membered cyclic N, O acetal in polybenzoxazine foam. Reproduced with permission [69]. Copyright 2025, Wiley‐VCH.

Ester bonds can undergo reversible reactions with hydroxyl groups through dynamic transesterification exchange following an associative mechanism (Scheme 5a). To facilitate this exchange without the addition of external catalysts, catalytic sites can be covalently incorporated into the polymer backbone. These internal catalytic centers, such as tertiary amines or functional groups capable of neighboring group participation (NGP), lower the activation barrier of the reversible process and enable dynamic exchange under practical processing conditions [21, 22].

Ester bonds are commonly introduced into polymer backbones via the polycondensation of polyacids and polyols, producing polyesters and water as a byproduct. Polyethylene terephthalate (PET), the most extensively produced commodity polyester, is obtained through this route. Like other thermoplastics, PET can be mechanically recycled above its melting temperature (250°C–260°C). However, such high processing temperatures promote chain scission, resulting in reduced mechanical strength and barrier properties in recycled materials [61]. To mitigate this issue, crosslinking agents or chain extenders can be added during reactive extrusion to restore the properties of recycled PET to levels comparable with virgin material. When dynamic chain extenders are used, they not only improve melt strength and foamability but can also enhance recyclability. Li and coworkers developed a one‐pot method to improve the melt strength, the foamability, and the dynamic capabilities of commercial PET [62, 63]. Their approach involved the ring‐opening of pyromellitic anhydride by a tertiary amine‐containing polyol to generate aromatic ester and carboxylic acid groups. The resulting ester linkages reacted reversibly with aliphatic hydroxyl groups, while tertiary amines and neighboring carboxylic acids facilitated dynamic transesterification exchanges (Scheme 5b). Reactive extrusion enabled in‐situ incorporation into PET, enhancing the melt strength and ensuring compatibility with Sc‐CO_2_ foaming. This approach enabled the production of low‐density PET foams (*ρ *=  39 kg·m^−3^) with large expansion ratios (Ф = 35) and closed‐cell morphologies.

The introduction of dynamic ester cross‐links has also inspired new strategies for designing various types of transesterification‐based DCPFs across various classes of polymers, including, polyurethane [55, 57], epoxy [64, 65, 66, 67], highly branched polyester [68], or polybenzoxazine [69]. Tian et al. utilized 1,5‐diaminopentane (PDA) activated with CO_2_ to form ammonium carbamate (PDAC), which acted as a latent blowing and crosslinker agent in epoxy‐based DCPFs [67]. Upon thermal decomposition, CO_2_ release regenerated the amine hardener, promoting ring‐opening polymerization (ROP) of epoxidized maleopimaric anhydride. By tuning the PDA/PDAC ratio, foams with average pore size down to 176 µm, porosities above 81%, and compressive strengths up to 3 MPa were obtained. Dynamic exchange of β‐hydroxyester bonds, internally catalyzed by tertiary amines, endowed these foams with self‐healing and degradability capabilities (recovery rate of 84% of the compressive strength compared to the original sample). In a related catalyst‐free approach, Sarrafan et al. designed dynamic epoxy‐based syntactic foams, i.e., lightweight composites reinforced with hollow glass microspheres, via the ROP of diglycidyl 1,2‐cyclohexane‐dicarboxylate with low‐molecular‐weight branched polyethyleneimine [64, 66]. In PUFs, ester‐containing segments have been incorporated using poly(*ε*‐caprolactone) (PCL)‐based diols [57]. These chemically blown thermosetting foams exhibited enhanced thermal and dimensional stability and could be converted into auxetic foams, i.e. foams characterized by a negative Poisson's ratio that contract laterally upon axial compression, through transesterification reactions.

Combining condensation‐induced self‐foaming with DCBs, Verge et al. developed a strategy for producing self‐foaming reprocessable polybenzoxazine foams [69]. Benzoxazine monomers containing alkyl ester and β‐aminoalcohol functionalities were synthesized in a two‐steps condensation process [70]. Upon thermal activation, ROP of the benzoxazine rings generated tertiary amines that served as internal catalytic centers for the NGP‐assisted transesterification between β‐aminoalcohol and alkyl ester groups (Scheme 5c). The irreversible transesterification of alkylester resulted in releasing alcohol as gas‐phase blowing agent. By tuning precursor functionality and blowing agent volatility, thermosetting foams with open‐cell structures, densities of 161–335 kg·m^−3^, and Young moduli between 3 and 38 MPa were obtained. Foam‐to‐film reprocessing was demonstrated, effectively closing the recycling loop for this distinctive class of self‐foaming DCPFs.

#### 3.2.4 Disulfide‐Based DCPFs

**SCHEME 6:**
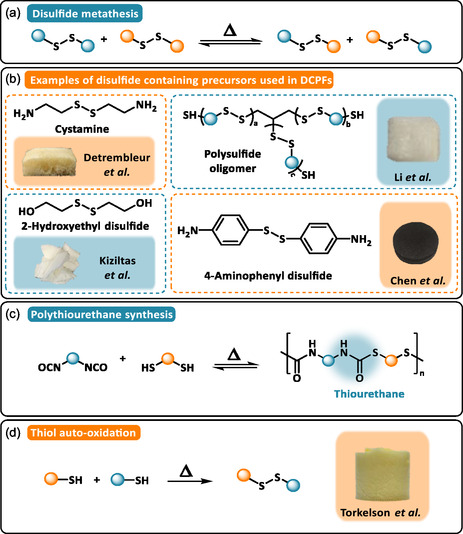
(a) General reaction mechanism of disulfide exchange. (b) Examples of disulfide‐containing precursors used in disulfide‐based DCPFs: Cystamine. Reproduced with permission [72]. Copyright 2025, Elsevier. 2‐Hydroxyethyl disulfide. Reproduced with permission [71]. Copyright 2022, American Chemical Society. Polysulfide oligomer. Reproduced with permission [58]. Copyright 2021, Royal Society of Chemistry. 4‐Aminophenyl disulfide. Reproduced with permission [75]. Copyright 2023, American Chemical Society. Synthetic approach for (c) polythiourethane and (d) mechanism for thiol auto‐oxidation producing disulfide bond. Reproduced with permission [73]. Copyright 2024, Elsevier.

Disulfide bonds are dynamic linkages with moderate bond dissociation energies and multi‐stimuli responsiveness (e.g. thermal, redox, or photoactivation), making them particularly attractive for the design of fast‐responsive DCPNs (Scheme 6a). Depending on the activation mode, disulfide exchange can proceed under catalyst‐free conditions via thiolate anion intermediates or through homolytic cleavage. As disulfide exchange does not need a catalyst to occur, a straightforward approach to benefit from this exchange consists of simply using building blocks that intrinsically contain the disulfide bonds (Scheme 6b).

Thiol‐terminated polysulfide oligomers (PSO) has been used as disulfide‐based soft segments in the fabrication of malleable and thermally recyclable water‐blown PUFs [58, 59]. Partial replacement of the telechelic aliphatic hydroxyl groups of polypropylene glycol (PPG) with reactive thiol groups from PSO enables disulfide incorporation via thiol–isocyanate click reactions forming thiourethane linkages (Scheme 6c). At relatively low PSO content (PPG:PSO  ≥  70:30  wt%), the resulting PUFs retain similar morphology, density, and mechanical resilience as conventional systems. Higher PSO contents require increased proportions of rigid aromatic isocyanates such as toluene diisocyanate [58] or polyisocyanate [59] to maintain stoichiometric balance. These disulfide‐containing PUFs undergo rapid stress relaxation through heat‐induced topological rearrangements, enabling foam‐to‐film reprocessing within minutes. The reprocessed films retain similar cross‐linking density (gel fractions > 89%) [58] and mechanical performance (*E* = 269 MPa) [59] after being reprocessed up to three times, evidencing the superior recycling efficiency and long‐term durability. Zhang and coworkers further demonstrated that introducing disulfide into the hard segments of PUFs enhances recyclability [71]. In their work, 2‐hydroxyethyl disulfide (HEDS) diol served as a co‐chain extender in flexible water‐blown PUFs. The inclusion of HEDS altered the curing kinetics and foaming process, improving cell uniformity and reducing pore size (100–500 μm). Notably, the long linear chain of HEDS interrupted the bulking of the aromatic‐MDI segments and lowered the glass transition temperature of the hard domains from *T*_g_ > 200°C to *T_g_* = 139°C–161°C. The chain mobility was improved, facilitating the reprocessing at lower temperatures and reducing the risk of depolymerization linked to dissociative urethane exchange. The promoted chain mobility facilitates reprocessing at lower temperatures and reduces the risk of depolymerization associated with dissociative urethane exchange. Detrembleur and coworkers expanded this strategy by using bio‐based cystamine to incorporate dynamic disulfide linkages into water‐blown NIPUFs [72]. While PHU‐based foams typically require a temperature ranging from 150°C to 180°C to promote dynamic exchange either through associative transcarbamoylation or reversible cyclic carbonate aminolysis, the incorporation of disulfide bonds reduced the network rearrangement window to 90°C–120°C.

Another disulfide‐incorporation synthetic pathway exploits the auto‐oxidation of pendant thiol groups (Scheme 6d). Torkelson and coworkers explored the fast aminolysis of cyclic dithiocarbonates (DTCs) to synthesize recyclable non‐isocyanate polythiourethanes (NIPTUs) foams [73]. Unlike NIPUs, NIPTU backbones contain thiocarbonyl and free thiol groups arising from the regioselective addition of primary amines to DTCs. Upon exposure to oxygen at 80°C, these thiols undergo gradually auto‐oxidation to form disulfide crosslinks. By varying the blowing agent (e.g. acetone or ethyl acetate) and DTC functionality (di‐ vs. trifunctional), closed‐cell NIPTU foams with low‐density and tunable properties were obtained (*ρ *=  140–300 kg·m^−3^, *T*_g_ = 9°C–21°C). The catalyst‐free disulfide exchange endowed these foams with extrudability, foam‐to‐film and foam‐to‐foam reprocessing, broadening their recycling potential.

These studies highlight that integrating dynamic disulfide bonds into PUFs significantly enhances reprocessability while reducing the temperature and time for network rearrangement. Exchange kinetics are further accelerated when sulfur atoms are conjugated with aromatic rings, where electron‐withdrawing substituents stabilize reactive intermediates and lower activation energies [74]. This approach has been leveraged in recyclable solar‐thermal evaporators for seawater desalination [75]. In this system, 4‐Aminophenyl disulfide (4‐AFD) was used as a disulfide‐containing amine hardener in the ROP of diglycidyl ether of bisphenol A (DGEBA). Composites foams reinforced with carbon nanotubes were fabricated via a cost‐effective NaCl particulate‐leaching method, yielding open and interconnected porous structures. The aromatic disulfide bonds endowed reprocessability, providing a sustainable recycling route for advanced functional materials.

#### 3.2.5 Imine‐Based DCPFs

**SCHEME 7:**
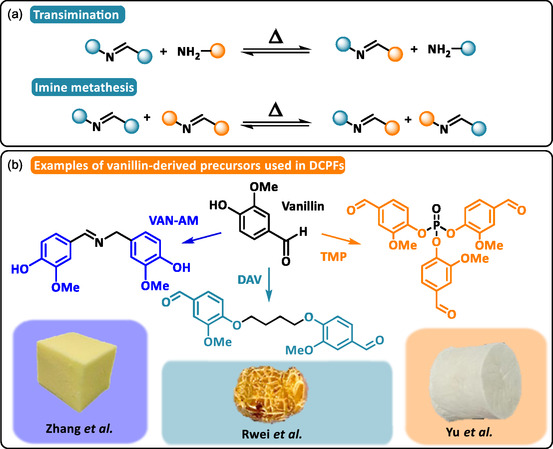
(a) General reaction mechanism of transimination and imine metathesis. (b) Examples of vanillin‐derived precursors used in imine‐based DCPFs: vanillin‐based diphenol obtained via the condensation of vanillin with vanillylamine (VAN‐AM). Reproduced with permission [60]. Copyright 2024, Elsevier. Vanillin‐based dialdehyde obtained via the Friedel‐Craft alkylation of vanillin with bromobutane (DAV). Reproduced with permission [76]. Copyright 2022, American Chemical Society. Vanillin‐based trialdehyde obtained via the condensation of vanillin with phosphorous oxychloride (TMP). Reproduced with permission [77]. Copyright 2024, American Chemical Society.

Imine bonds are readily formed under mild conditions through the condensation of primary amines with aldehyde or ketone carbonyl groups. Also known as Schiff bases, these linkages can undergo reversible exchange either with primary amines (transimination) or between existing imines (imine exchange) following an associative mechanism (Scheme 7a). In addition to their rapid exchange kinetics, often occurring at temperatures as low as 50°C, imine bonds are highly sensitive to pH conditions, enabling controlled hydrolytic degradation.

Vanillin, a bio‐derived aldehyde obtained by the catalytic oxidation of lignin, has been widely used to design imine‐based DCPNs and, by extension, DCPFs (Scheme 7b) [60, 76, 77]. Using a vanillin‐derived dialdehyde, Chen et al. synthesized poly(imine‐amide) networks capable of microcellular foaming [76]. In their approach, imine bonds were generated through cross‐linking reactions between the dialdehyde and pendant amine groups from linear or branched polyamides. Sc‐CO_2_ batch foaming produced uniform foams with low densities (*ρ *≈  100 kg·m^−3^) and high expansion ratios (Ф > 12). However, the expansion ratio decreased from 12.1 to 2.2 along with the foaming temperature increase from 150°C to 160°C. This reduction of the foaming efficiency is most likely due to rapid dynamic exchanges, with characteristic stress relaxation times (τ*) below 10 s at 110°C–150°C. It resulted in reduced viscoelastic performance and creep‐induced instability of the cellular structure, rendering it unable to retain its morphology. This observation highlights the need to carefully optimize foaming conditions and degree of cross‐linking to preserve the microcellular structure. To increase the density of dynamic cross‐links, He et al. synthesized a polyaldehyde precursor by reacting vanillin with phosphoryl chloride, which was subsequently cross‐linked with polyfunctional amines [77]. Ultralight aerogels were obtained by freeze‐drying technique, which exhibited honeycomb‐like morphologies and densities as low as 55 kg·m^−3^. To mitigate the inherent moisture sensitivity of imine bonds, hydrophobic fluorinated moieties were incorporated into the network, imparting superhydrophobicity (*θ *≈  155°) and enhanced hydrolytic stability. Owing to the high imine content, these networks could be rapidly depolymerized in acidic media within minutes, producing soluble monomers and oligomers that were fully recoverable and reprocessable into recycled aerogels. Comparable dynamic covalent aerogels have also been synthesized from petroleum‐based terephthalaldehyde precursors [78].

Beyond hydrolytic sensitivity, imine‐based DCPNs are often designed with *T*_g_ above the operational window of the dynamic exchange to maintain structural integrity under service conditions. Wu et al. demonstrated this concept by confining imine linkages within the hard domains of PUFs to regulate dynamic exchange kinetics [60]. A rigid aromatic polyol containing imine bonds was prepared via condensation of vanillin with vanillylamine which was subsequently incorporated into a PU matrix. The dynamic imine bonds remained inactive below the *T*_g_ of the hard domains (*T*_g_ = 113°C). Above this threshold, segmental motion activates reversible imine exchange, thus enabling foam‐to‐film reprocessing. Reprocessed films exhibit similar *T*_g_ and gel content (*T*_g_ ≈ 80°C, GC = 90%), confirming retention of the cross‐linking density.

Overall, the integration of dynamic imine bonds into polymer foams offers a versatile route for developing adaptive, recyclable, and degradable materials. Through rational molecular design, imine‐based DCPFs achieved a tunable balance between durability and reprocessability while maintaining structural integrity under operating conditions.

#### 3.2.6 Boronic Ester‐Based DCPFs

**SCHEME 8:**
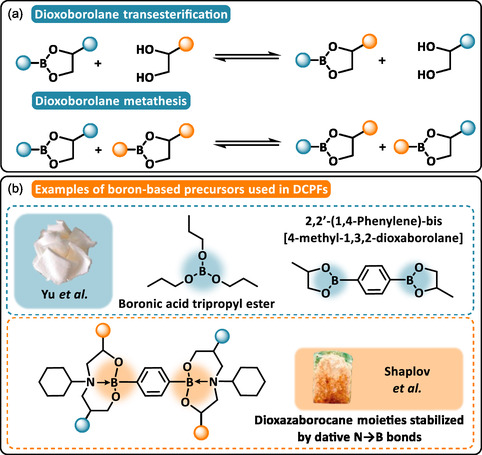
(a) General reaction mechanism of dioxaborolane metathesis and transesterification. (b) Examples of boron‐based precursors used in boronic ester‐based DCPFs: boronic acid tripropyl ester (BATTE) and 2,2′‐(1,4‐Phenylene)‐bis [4‐methyl‐1,3,2‐dioxaborolane] (PBMDB). Reproduced with permission [81]. Copyright 2021, Elsevier. Dioxazaborocane moieties stabilized by dative N → B bond. Reproduced with permission [83]. Copyright 2025, American Chemical Society.

Boronic esters and their cyclic analogs, dioxaborolanes, are dynamic linkages formed through the esterification of boronic acids with alcohols. Reversible transesterification with diols [79] or metathesis‐type exchange [80] exhibit rapid dynamic equilibria at relatively low‐temperature (Scheme 8a). Although their inherent moisture sensitivity may affect dimensional stability and long‐term performance, recent engineering studies have been developed to mitigate these issues.

In DCPFs, boronic ester linkages have been integrated into linear thermoplastics via two main cross‐linking strategies: (i) transesterification between boronic esters and alkyl esters [81] and (ii) direct esterification of diboronic acids with pendant hydroxyl groups [82, 83]. In the first approach, Yu et al. examined the effect of various boron‐containing cross‐linkers on the network topology and foaming behavior of ethylene–vinyl acetate (EVA) copolymers [81]. A model reaction confirmed the dynamic reactivity of triethyl borate with methyl methacrylate using tetrabutyl titanate catalyst. Building on this, they prepared boronic ester or dioxaborolane‐containing EVA foams by reacting boronic acid or boronic ester precursors (Scheme 8b). The functionality of the cross‐linker strongly influenced foam properties with an increased gel content resulting in reduced cell size and increased Young modulus. The reversible nature of the boronic ester network allowed repeated transitions between bulk and foamed states, demonstrating the recyclability of EVA‐based DCPFs. Following the second strategy, Huang et al. synthesized recyclable foams by esterifying a diol‐containing styrene copolymer with 1,4‐phenylenediboronic acid [82]. The resulting foams, fabricated via hot‐pressing/salt‐leaching foaming process, exhibited interconnected hierarchical porosity and superhydrophobicity (*θ*_H2O_ = 154°). In a similar approach, Shaplov and coworkers used diboronic acid precursors to cross‐link proximal diol and hydroxyl groups in linear thermoplastic PHUs forming, respectively, dioxaborolane or dioxazaborocane moieties (Scheme 8b) [83]. They demonstrated that nitrogen‐stabilized dioxazaborocane structures, formed through dative N → B interactions, significantly reduced moisture sensitivity while maintaining the mechanical performance under humid conditions. Lightweight foams with densities as low as 70 kg·m^−3^ were obtained via vacuum compression molding using ethanol as a physical blowing agent, confirming the suitability of these systems for foaming applications.

In summary, despite the intrinsic hydrolytic lability of boronic esters, recent molecular engineering approaches have enabled the development of DCPFs that retain stability under humid conditions. To date, most studies have focused on thermoplastic systems. Extending these strategies to thermosetting matrices could further expand the applicability of boronic ester–based DCPFs toward more robust, recyclable, and self‐healable foams.

#### 3.2.7 Carbonate‐Based DCPFs

**SCHEME 9:**
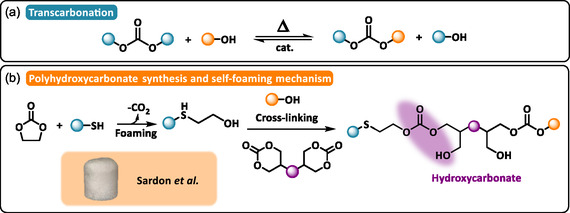
(a) General reaction mechanism of transcarbonation. (b) Synthetic approach for hydroxypolycarbonate and self‐foaming mechanism through decarboxylative S‐alkylation of five‐membered cyclic carbonate. Reproduced with permission [85]. Copyright 2023, Wiley‐VC.

Analogous to the transesterification of ester bonds, transcarbonation between carbonate linkages and hydroxyl groups proceeds through an associative exchange mechanism (Scheme 9a). These reversible linkages, commonly present in polycarbonates and CO_2_‐based polymers, can undergo bond reshuffling in the presence of suitable catalysts [84].

In polymer foams, thiol‐induced decarboxylation of cyclic carbonates has been exploited to generate self‐foaming NIPUFs with reprocessing capabilities through transcarbamoylation or reversible aminolysis reactions [14]. When polyamines are omitted from the formulation, a polycarbonate network containing permanent β‐hydroxythioether bonds is formed. Sardon and coworkers explored the chemoselective reactivity of cyclic carbonates toward thiols to design self‐foaming polycarbonates that are both mechanically reprocessable and chemically recyclable [85]. 1,8‐Diazabicycloundec‐7‐ene (DBU)‐catalyzed model studies revealed that nucleophilic thiol addition proceeds selectively with five‐membered cyclic carbonates, whereas six‐membered analogs display negligible reactivity under comparable conditions. Interestingly, the opposite trend was observed for the alcoholysis of cyclic carbonates containing β‐hydroxythioether bonds (Scheme 9b). This chemoselectivity was leveraged in the one‐pot cascade ROP of five‐ and six‐membered cyclic carbonates, initiated by thiols and β‐hydroxythioethers bonds, respectively, to produce self‐blown foams under mild conditions (70°C, 1 h). Optimization of precursor stoichiometry and foaming parameters yielded resilient thermosetting foams with densities ranging from 180 to 278 kg·m^−3^. The dynamic nature of carbonate exchanges enabled efficient foam‐to‐film reprocessing through thermally activated exchanges (GC = 83% and 87% for the foam and the film, respectively), while their susceptibility to acid‐catalyzed cleavage facilitated chemical recycling routes analogous to industrial depolymerization processes.

The design of carbonate‐based DCPFs can be further tuned using bio‐based polyols as chain extenders [86]. Increasing the thiol functionality or enhancing the rigidity of substituents on the cyclic carbonate moiety promotes higher cross‐linking densities, thereby improving both mechanical performance and thermal stability.

#### 3.2.8 Vinylogous Urethane‐Based DCPFs

**SCHEME 10:**
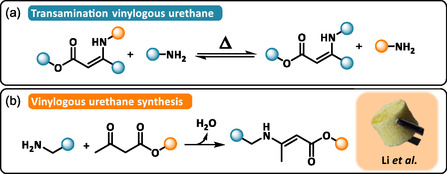
(a) General reaction mechanism of transamination of vinylogous urethane. (b) Synthetic approach for vinylogous urethane from the condensation of primary amine and acetoacetate. Reproduced with permission [88]. Copyright 2024, Elsevier.

Vinylogous urethane (VU) bonds are typically formed via the straightforward condensation between ketoesters and primary amines. VU bonds can undergo associative transamination reactions at elevated temperatures (>100°C–120°C) under catalyst‐free conditions (Scheme 10a) [87]. In recent years, vinylogous urethane‐based DCPNs have gained increasing attention due to their combination of robust mechanical properties, characteristic of conventional urethanes, with exceptional dynamic adaptability.

Li et al. recently reported the first example of a closed‐loop recyclable poly(vinylogous urethane) thermoset foam [88]. Their approach employed a cryo‐polymerization technique using dimethyl sulfoxide as a pore‐forming agent to generate a cross‐linked vinylogous urethane network through amino‐acetoacetate polycondensation (Scheme 10b). Following solvent removal, the resulting DCPFs exhibited a sponge‐like macroporous morphology with densities ranging from 170 to 420 kg·m^−3^, excellent mechanical resilience, and thermal stability up to 260°C. A defining feature of this system is the selective acid‐catalyzed hydrolysis of vinylogous urethane bonds, which enables recovery of the original amine and acetoacetate monomers after appropriate separation and purification. These recovered building blocks were successfully reused to re‐synthesize poly(vinylogous urethane) DCPFs, thereby demonstrating a truly closed‐loop lifecycle. The higher compressive strength measured for the refoamed material (+30% compared to the original foam) was attributed to the higher purity of the recovered monomers leading to a denser cross‐linked network.

#### 3.2.9 DCPFs Relying on Other Dynamic Chemistries

**SCHEME 11:**
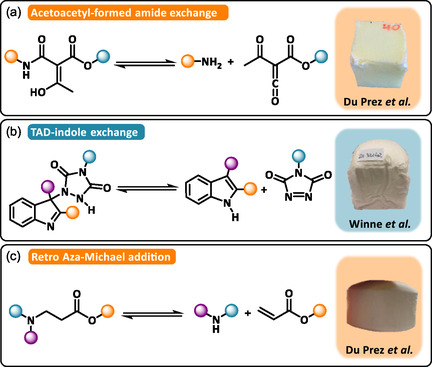
General reaction mechanism of (a) Acetoacetyl‐formed amide exchange. Reproduced with permission [89]. Copyright 2023, American Chemical Society. (b) Triazolinedione (TAD)‐indole exchange. Reproduced with permission [90]. Copyright 2024, American Chemical Society. (c) Retro Aza‐Michael addition. Reproduced with permission [91]. Copyright 2024, Wiley‐VCH.

A series of dissociative dynamic covalent bonds have also been explored to impart reprocessing capabilities to polymeric foams. Du Prez and coworkers further advanced the sustainability of PUFs by integrating various dissociative dynamic bonds to achieve both elastomeric reprocessing [89, 90] and foam regeneration [91]. Among these systems, isocyanate–acetoacetate adducts were shown to form dynamic β‐ketoamide motifs capable of reversible dissociation into amines and ketenes above 80°C (Scheme 11a) [89]. In a multistep design, thermoreversible triazolinedione (TAD)–indole adducts were introduced into the polyol backbone of PUFs (Scheme 11b) [90]. An alternative strategy involved β‐aminoester bonds generated via the reversible Aza–Michael addition between acrylates and amines (Scheme 11c) [91]. These approaches, fully compatible with standard PUF formulations and foaming protocols, yielded recyclable DCPFs with morphology and mechanical properties comparable to conventional PUFs. Moreover, the incorporation of these type of dynamic bonds effectively lowered the network rearrangement temperature, mitigating the depolymerization risks typically associated with urethane bond cleavage at elevated temperatures.

Table 2 summarizes the properties of several examples of DCPFs containing various type of reversible bonds.

**TABLE 2:** Selected examples of dynamic exchange reactions based on dissociative and associative mechanism incorporated in dynamic covalent polymeric foams.

Polymer foam (blowing mechanism)	Foam characteristics	Foam properties	Dynamic exchange (catalyst)	New dynamic functionalities	References
Polyamide (physical)^a^	*ρ *= ∅^b^ Ф = 3.1–16.9^c^ ȼ = 15–24 µm^d^	Flexible foam *T*_g_ = ∅^e^ *T*_d5%_ = ∅^f^	Diels–Alder (catalyst‐free)	Foam‐to‐foam recycling	[44]
Polyurethane (physical)^g^	*ρ *= 36–38 kg·m^−3^ Ф = ∅ ȼ = 310–410 µm	*σ *= 0.2–0.3 MPa^h^ *T*_*α*_ = −4°C to 1°C^i^ *T*_d10%_ = 239°C–252°C^j^	Transcarbamoylation (catalyst‐free)	Foam‐to‐film reprocessing	[49]
Non‐isocyanate polyurethane (self‐foaming)^k^	*ρ *= 167–310 kg·m^−3^ Ф = ∅ ȼ = 0.5–1.5 mm	*E* = 1–211 kPa^l^ *T*_g_ = −25°C–43°C *T*_d5%_ = 231°C–237°C	Transcarbamoylation and reversible cyclo‐carbonate aminolysis (DBU^m^ or catalyst‐free)	Foam‐to‐film reprocessing	[52]
Epoxy (chemical)^n^	*ρ *= 138–182 kg·m^−3^ Ф = 5.5–6.6 *e* = 80%–85%^o^	*E* = 40 MPa *T*_g_ = 70°C *T*_d5%_ = 201°C–239°C	Transesterification (catalyst‐free)	Self‐healing and degradability	[67]
Benzoxazine (self‐foaming) p	*ρ *= 161–335 kg·m^−3^ Ф = 2.2–8.5 *e* = 67%–86%	*E* = 3–38 MPa *T*_g_ = ∅ *T*_d5%_ = 217°C–244°C	Transesterification (catalyst‐free)	Self‐foaming and foam‐to‐film reprocessing	[69]
Polyurethane‐co‐thiourethane (chemical)^q^	*ρ *= 58–78 kg·m^−3^ Ф = ∅ ȼ = 300–375 µm	Flexible foam *T*_*α*_ = 29°C–77°C *T*_d5%_ = 242°C–267°C	Disulfide exchange (catalyst‐free)	Reshaping and foam‐to‐film reprocessing	[59]
Non‐isocyanate polyurethane (chemical)^r^	*ρ *= 232–379 kg·m^−3^ Ф = ∅ ȼ = 0.3–1 mm	Flexible foam *T*_g_ = 15°C–30°C *T*_d5%_ = 239°C–254°C	Transcarbamoylation and reversible cyclo‐carbonate aminolysis and disulfide exchange (DBU or catalyst‐free)	Foam‐to‐film reprocessing	[72]
Polyimine (physical)^s^	*ρ *= 113–121 kg·m^−3^ Ф = ∅ ȼ = 0.5–0.6 µm	*E* = 0.2–0.3 MPa T_g _= ∅ *T*_d _= ∅	Imine exchange (catalyst‐free)	Self‐healing and foam‐to‐foam recycling	[78]
Polystyrene (physical)^t^	*ρ *= 370–590 kg·m^−3^ Ф = ∅ *e* = 44%–65%	*E* = ∅ *T*_g_ = 102°C *T*_d_ = 364°C	Transesterification boronic ester (catalyst‐free)	Foam‐to‐foam recycling	[82]
Polycarbonate (self‐foaming)^u^	*ρ *= 180–278 kg·m^−3^ Ф = ∅ ȼ = 0.7–1 mm	Flexible foam *T*_g_ = −16°C to −14°C *T*_d5%_ = 115°C–121°C	Transcarbonation (DBU)	Degradability and foam‐to‐film reprocessing	[85]
Poly(vinylogous urethane) (physical)^v^	*ρ *= 170–420 kg·m^−3^ Ф = ∅ ȼ = 14–32 µm	Flexible foam *T*_g _= ∅ *T*_d5%_ = 263°C–281°C	Vinylogous urethane exchange (catalyst‐free)	Foam‐to‐foam recycling	[88]
Polyurethane (chemical)^w^	*ρ *= 90–150 kg·m^−3^ Ф = ∅ ȼ = 0.8–1 mm	Flexible foam *T*_g_ = 14°C–23°C *T*_d5%_ = 245°C–259°C	Retro Aza‐Michael addition (catalyst‐free)	Foam‐to‐film reprocessing and foam‐to‐foam recycling	[91]

a Prepared from the DA‐cycloaddition between 1,1′‐(methylenedi‐4,1‐phenylene) bismaleimide and polyamide copolymer of 2,5‐furandicarboxylic acid, 11‐aminoundecanoic acid, and dimer diamine.

b Foam density.

c Expansion ratio defined as the ratio of the final foam volume to the initial liquid/premix volume.

d Average cell size.

e Glass transition temperature determined from DSC or TMA experiment.

f Temperature of 5% weight loss.

g Prepared from a cardanol‐based phenol obtained from the Friedel–Crafts alkylation of cardanol with guaiacol, polylactide diol, and polymethylene polyphenyl polyisocyanate.

h Compression strength.

i α‐mechanical relaxation temperature determined from DMA experiment.

j Temperature of 10% weight loss.

k Prepared from trimethylpropane triscarbonate, thiolactone, 1,2‐bis(2‐aminoethoxy) ethane, and m‐xylylene diamine

l Young modulus.

m 1,8‐diazabicyclo [5.4.0]undec‐7‐ene.

n Prepared from epoxidized maleopimaric anhydride and 1,5‐diaminopentane.

o Porosity.

p Prepared from alkylester‐based benzoxazine monomer end‐capped with β‐aminoalcohol group.

q Prepared from polypropylene glycol, polysulfide oligomer, and polyisocyanate.

r Prepared from trimethylolpropane triglycidyl carbonate, cystamine, and/or hexamethylenediamine.

s Prepared from terephthalaldehyde, diethylenetriamine, and tris(2‐aminoethyl)amine.

t Prepared from styrene/4‐(2,2‐dimethyl‐1,3‐dioxolan‐4‐yl) butyl methacrylate copolymer cross‐linked with 1,4‐benzenediboronic acid.

u Prepared from pentaerythritol tetrakis, poly(caprolactone) triol, di(trimethylolpropane carbonate), and ethylene carbonate.

v Prepared from hexamethylene diacetoacetate, tris(2‐aminoethyl)amine, and 1,3‐bis(3‐aminopropyl)tetramethyldisiloxane.

w Prepared from a β‐aminoester polyol, Pripol 2034 diol, pentaerythritol ethoxylate, and toluene diisocyanate.

## 4 Dynamic Exchange Reactions as Promoters of the Next‐Generation of Polymer Foams?

Dynamic covalent chemistry (DCC) was initially adopted as a design concept in polymer foams to enable network rearrangement and to introduce new end‐of‐life opportunities. Nowadays, their role in polymer foams has evolved significantly. Indeed, DCBs are now also considered as molecular tools that can be used to influence foam formation, architecture, and durability. Rather than being limited to post‐use recovery, DCBs are also used as a proactive design principle across the entire lifecycle of polymer foams. The following sections highlight how dynamic exchange reactions can be strategically activated during foam processing, service, and end‐of‐life to program material behavior.

### 4.1 Dynamic Covalent Chemistry as a Design Lever for Foams Architecture

Similarly to conventional polymer foams, DCPFs are generally produced by formulating precursors with a blowing agent. Foaming proceeds through cell nucleation, growth, and stabilization, which collectively govern the final morphology and the mechanical properties of the foam. The increase of the viscosity during network formation is a critical processing parameter as it must be balanced to enable foam expansion while preventing cell coalescence or collapse. However, unlike conventional polymer foams, DCPFs incorporate dynamic covalent bonds capable of reversible exchange, expected to facilitate foam expansion as the dynamic exchanges can relax the stress applied by the blowing agents onto the cell wall during the growth step. Nevertheless, the kinetics of bond exchange reactions must be carefully synchronized with gas generation and cell stabilization to achieve a stable cellular structure. Consequently, the successful fabrication of DCPFs requires, in addition to conventional processing parameters, a fine control and anticipation of the effects of dynamic bonds onto the resulting cell morphology, specifically for DCPFs relaxing promptly an applied stress. Moreover, dynamic covalent reactions can be selectively activated during foaming to control cellular nucleation, tune matrix’ viscoelasticity, and enable self‐foaming through bond dissociation. This approach represents a paradigm shift in foam engineering: instead of designing dynamic bonds solely for recyclability, they now tend to be leveraged to tailor materials’ structure from the molecular level upward.

#### 4.1.1 Programming Foams Architecture

**FIGURE 3:**
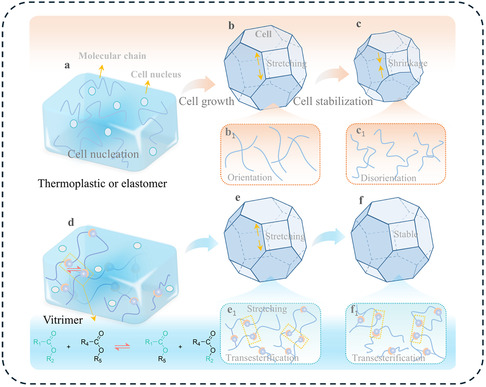
Regulation of the conformation and configuration in the foaming process of thermoplastic or elastomer (a–c) and dynamic covalent polymeric foam (d–f). Reproduced with permission [63]. Copyright 2023, Royal Society of Chemistry.

Optimization of the morphology of polymer foams is a subtle balance between the evolution of viscoelastic properties and gas release. The key challenge is to maintain a viscosity that is low enough to allow cell growth and expansion, yet high enough to maintain dimensional stability. During the cell nucleation stage, the cell walls experience intense stretching, leading to the accumulation of residual stress. DCPFs can release this stress through dynamic exchange reactions. However, in reversible networks that exhibit a strong temperature‐dependance such as dissociative DCPFs, the material lacks the mechanical strength required to withstand internal gas pressure above dissociation threshold [44]. It highlights the critical importance of tuning the kinetic of the dynamic bonds to align with the different foaming stages and to define optimal foaming conditions. At temperatures where the kinetic of the dynamic exchange is low, the dynamic network remains mechanically rigid, restricting polymer chain mobility, and prevents bubble nucleation and expansion. As the foaming temperature approaches the temperature of bond exchange, the softening of the matrix and the relaxing constraints on cell growth support the nascent cellular structure (Figure 3) [62, 63]. Leading to a more stable state, the configuration‐change process addresses the typical issues of shrinkage, cracks, and dimensional instability in thermoplastic or elastomeric‐based foams. In this regime, the dynamic network achieves an effective balance between structural support and mobility, enabling the formation of highly expanded foams with a uniform microcellular morphology. This function outlines the unique interplay of dynamic exchange reactions in controlling both the morphology and stability of durable DCPFs.

#### 4.1.2 Dynamic‐Assisted Self‐Blowing Mechanisms

**FIGURE 4:**
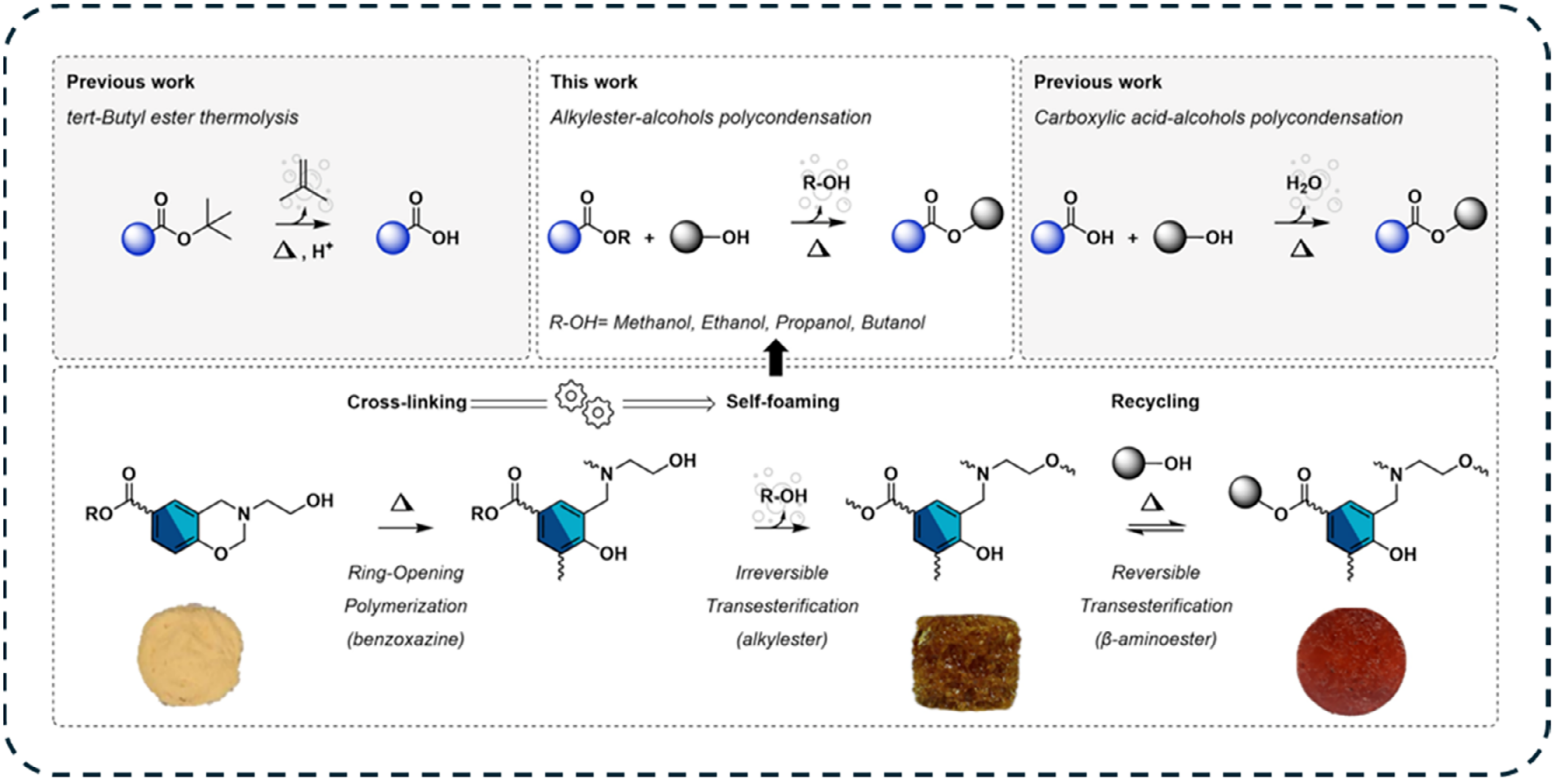
Mechanism of self‐blowing and mechanical reprocessing in transesterification‐based polybenzoxazine foam. Reproduced with permission [69]. Copyright 2025, Wiley‐VCH.

Another emerging strategy leverages dynamic exchange reactions as self‐blowing agent for the fabrication of recyclable foams [69, 92]. In these systems, foaming is induced by the dissociation of volatile species covalently attached to the polymer backbone, which are liberated during the dynamic exchange process [11]. Verge et al. demonstrated this approach in polybenzoxazine foams, where transesterification reaction generated alcohol as a volatile byproduct [69]. In these systems, benzoxazine ROP and transesterification occur simultaneously due to a mechanistic coupling between the two reactions. As a result, the foaming process occurs alongside the polybenzoxazine network formation. The molecular design of the precursor enabled the in situ formation of β‐aminoalcohol, which then catalyzed transesterification reactions through neighboring group participation effects (Scheme 5c) [70]. In this structure, the alcohol to be released is embedded as a short side‐chain alkyl ester. Once polymerization begins, the tertiary amine is formed and catalyzes the transesterification reactions, leading to the formation of the β‐aminoester and alcohol adducts (Figure 4). At the curing temperature of the precursors (180°C–220°C), the alcohol evaporates quickly and acts as a self‐blowing agent, promoting the formation of a cellular structure. Precursors containing different alkyl ester groups were synthesized, enabling the release of various alcohols ranging from methanol to butanol. The physical properties of these alcohols, particularly their boiling points and vapor pressures, had a strong influence on the foaming process. By combining these precursors with different processing conditions, foams with a wide range of expansion ratios were obtained. After completion of the curing and foaming processes, the resulting β‐aminoester linkages were also capable of undergoing reversible exchange with pendant hydroxyl groups, enabling foam‐to‐film reprocessing. In a similar dynamic self‐foaming approach, Xie and coworkers explored the dissociation of acetoxime‐based urethane linkage and subsequent acetoxime vacuum‐assisted evaporation to regenerate a foam from PUF waste [92].

The development of self‐foaming polymers presents a significant advancement in foam technology as it eliminates the need for external physical or chemical blowing agents. This simplifies formulation, reduces costs, and improves sustainability of the foaming processes. This approach provides precise control over gas generation that is intrinsically coupled with network formation, enabling uniform cell morphology and tunable expansion without additional additives.

### 4.2 Service‐Life Extension

During their operational lifetime, polymer foams inevitably experience structural degradation, including microcrack formation and cell wall collapse. These irreversible damages compromise mechanical integrity and functional performance, ultimately leading to premature replacement and disposal. The introduction of DCBs provides polymer foams with the capacity to adapt and recover their dimensional stability via topological network rearrangements. This additive‐free strategy imparts DCPFs with remarkable healing and reorganizational capabilities, improving the service‐life of polymer foams.

#### 4.2.1 Rejoining Foam Scraps

**FIGURE 5:**
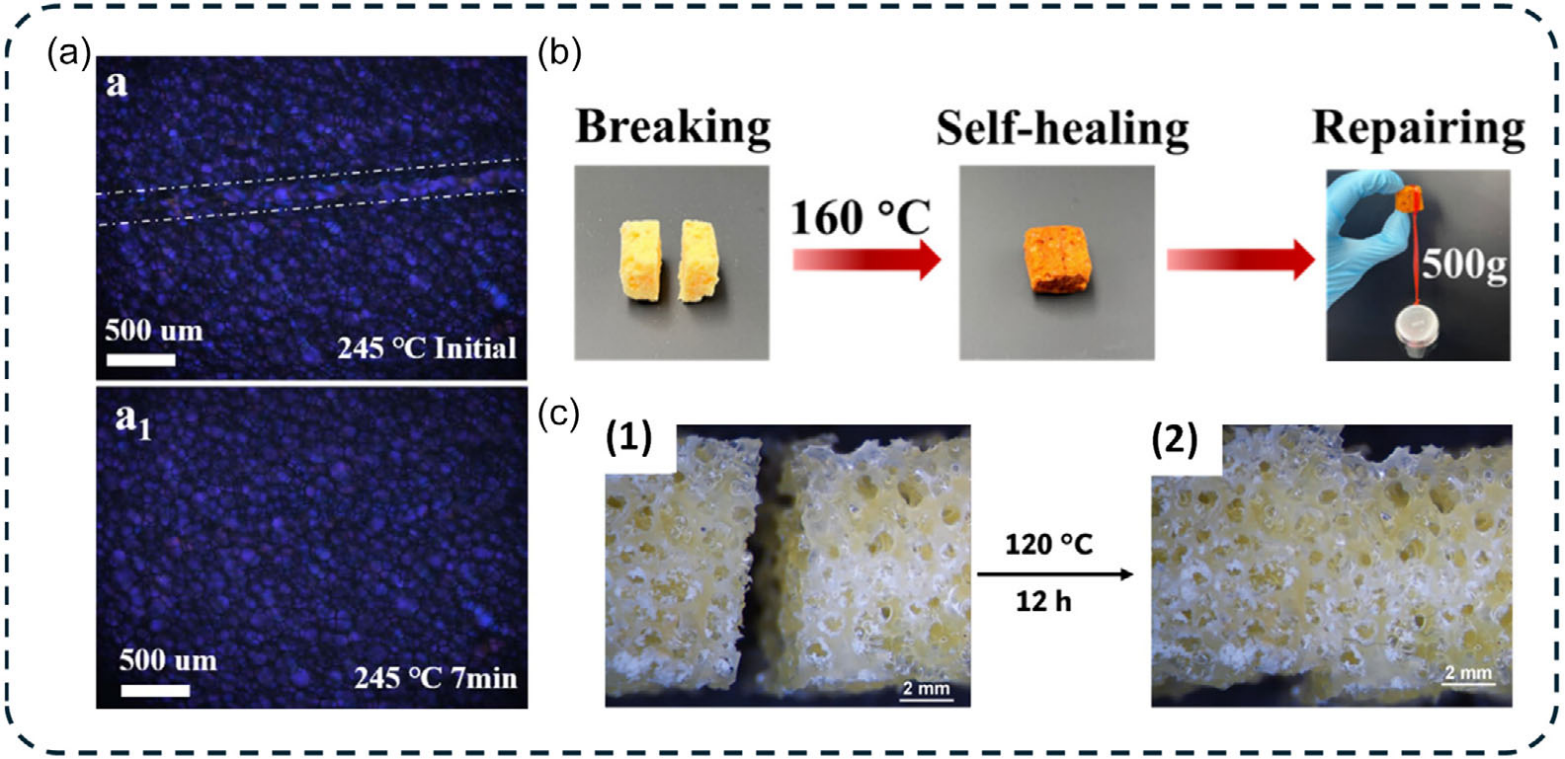
Examples of self‐healable dynamic covalent polymeric foam. Self‐healing behavior of: (a) Transesterification‐based PET foam observed by polarized optical microscopy. Reproduced with permission [63]. Copyright 2023, Royal Society of Chemistry. (b) Transesterification‐based epoxy foam. Reproduced with permission [67]. Copyright 2024, American Chemical Society. (c) Transcarbamoylation‐based NIPTU foam. Reproduced with permission [73]. Copyright 2024, Elsevier.

Rejoining foam scraps refers to the process of repairing or reconstructing polymer foams by fusing separate foam fragments into a single cohesive structure. This strategy enables the reuse of damaged or discarded foam pieces while preserving their cellular architecture and functional properties. In DCPFs, the process involves the thermal activation of the DCBs within the foam under pressure‐free conditions to prevent cell collapse. At the contact surface between foam fragments, dynamic exchange reactions promote autonomous healing of cracks [63, 68] (Figure 5a), resulting in the formation of a uniform welded foam block (Figure 5b,c) [65, 67, 68, 73, 81]. The efficiency of the rejoining process depends on both the kinetics of the dynamic exchange reactions and the quality of interfacial contact. A larger contact area promotes the density of exchange reactions and improves welding efficiency. Although rejoined DCPFs may not fully recover their original mechanical properties (recovery rate of nearly 84% of original compressive strength [67] or 50% of original elongation at break and tensile strength [73]), they retain sufficient structural integrity to withstand mechanical loading without failure [67, 68]. This dynamic adaptability is particularly advantageous in applications where polymer foams are routinely exposed to repetitive stress, enabling the recovery and reuse of damaged foams, overall minimizing lifecycle costs and environmental impact.

#### 4.2.2 Reorganization of the Cellular Structure

**FIGURE 6:**
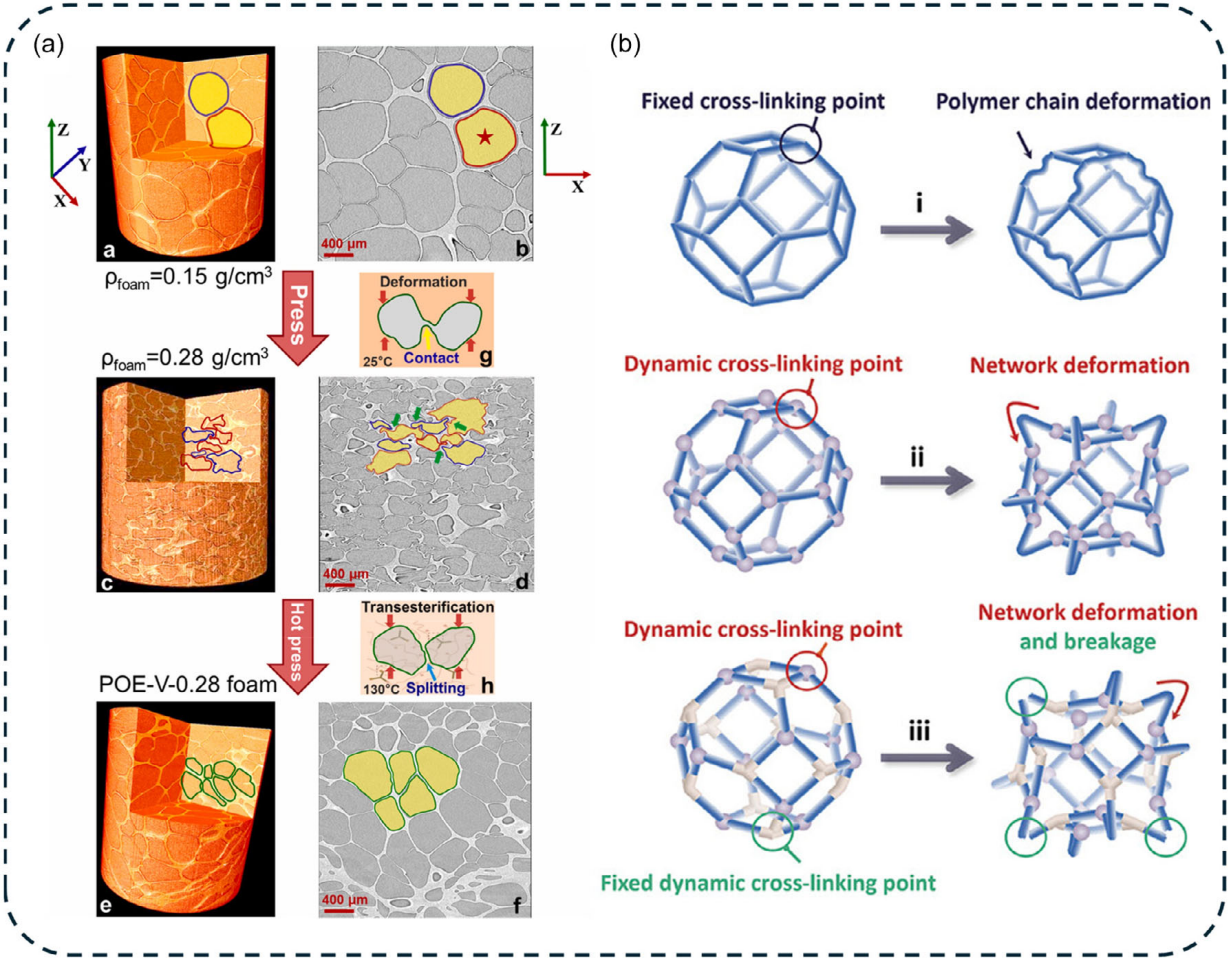
Examples of the reorganization of the cellular structure of dynamic covalent polymeric foam. (a) Micro‐computed X‐ray tomography images of the rearrangement process of transesterification‐based polyolefin DCPF during compression. Reproduced with permission [65]. Copyright 2021, Elsevier. (b) Transformation process of the foam network from circular open‐cell to re‐entrant cell when subjected to compression force. Reproduced with permission [59]. Copyright 2024, Wiley‐VCH.

In traditional foams, the permanent network restricts polymer chain mobility under mechanical stress, limiting their ability to readapt their three‐dimensional architecture. In contrast, DCBs enable stress redistribution and promote the reorganization of the foam’ cellular structure. Yu and coworkers examined the deformation of cell walls in transesterification‐based thermoplastic foams under unidirectional compression (*T* = 130°C, *t* = 1 h, *P* = 2 MPa) [65]. They observed that interfacial dynamic exchange reactions induced fusion between adjacent buckled cell walls and splitting of cells, leading to a reconfigured cellular morphology (Figure 6a). This ability to regulate cell structure demonstrates the potential of dynamic foams to achieve controlled densities and tunable mechanical properties. This reconfigurability has enabled the development of recyclable auxetic thermosetting foams [57, 59]. Hu and colleagues proposed a model describing stress distribution and network deformation in auxetic DCPFs, highlighting the role of dynamic cross‐linking points in facilitating local deformation and continuous network rearrangement (Figure 6b). In both transesterification‐based PUFs [57] and disulfide‐based polyurethane‐co‐thiourethane [59], stress relaxation induced by dynamic exchange reactions transforms the original open‐cell morphology into re‐entrant cells with inward‐pointing angles. When subjected to uniaxial [59] or triaxial [57] compression, these re‐entrant cells impart isotropic auxeticity to the thermosetting foams. Overall, the unique structural reconfigurability of DCPFs enables precise control of the microstructure, and paves the way toward durable and high‐performance foams with enhanced mechanical resilience.

### 4.3 Closing the Loop of the Lifecycle

Unlike thermoplastics, which can be re‐melt and reshaped, thermosetting foams possess a permanent crosslinked network that prevents their melt‐recycling and limits their reuse in high value second‐life applications. Although numerous strategies have been explored to valorize post‐consumer foams, only a few succeeded preserving the original material properties without causing irreversible degradation [3]. DCBs offers a transformative solution by enabling reversible bond exchange, allowing foams to be reprocessed or regenerated in a sustainable manner. Two main closed‐loop recycling strategies have recently emerged: (i) mechanical reprocessing into dense bulky polymers (*foam‐to‐film reprocessing*) and (ii) regeneration of the cellular architecture (*foam‐to‐foam recycling*). The key challenge of these closed‐loop recycling strategies lies in producing secondary materials that maintain structural integrity and mechanical properties comparable to the original foam.

#### 4.3.1 Foam‐to‐Film Reprocessing

**FIGURE 7:**
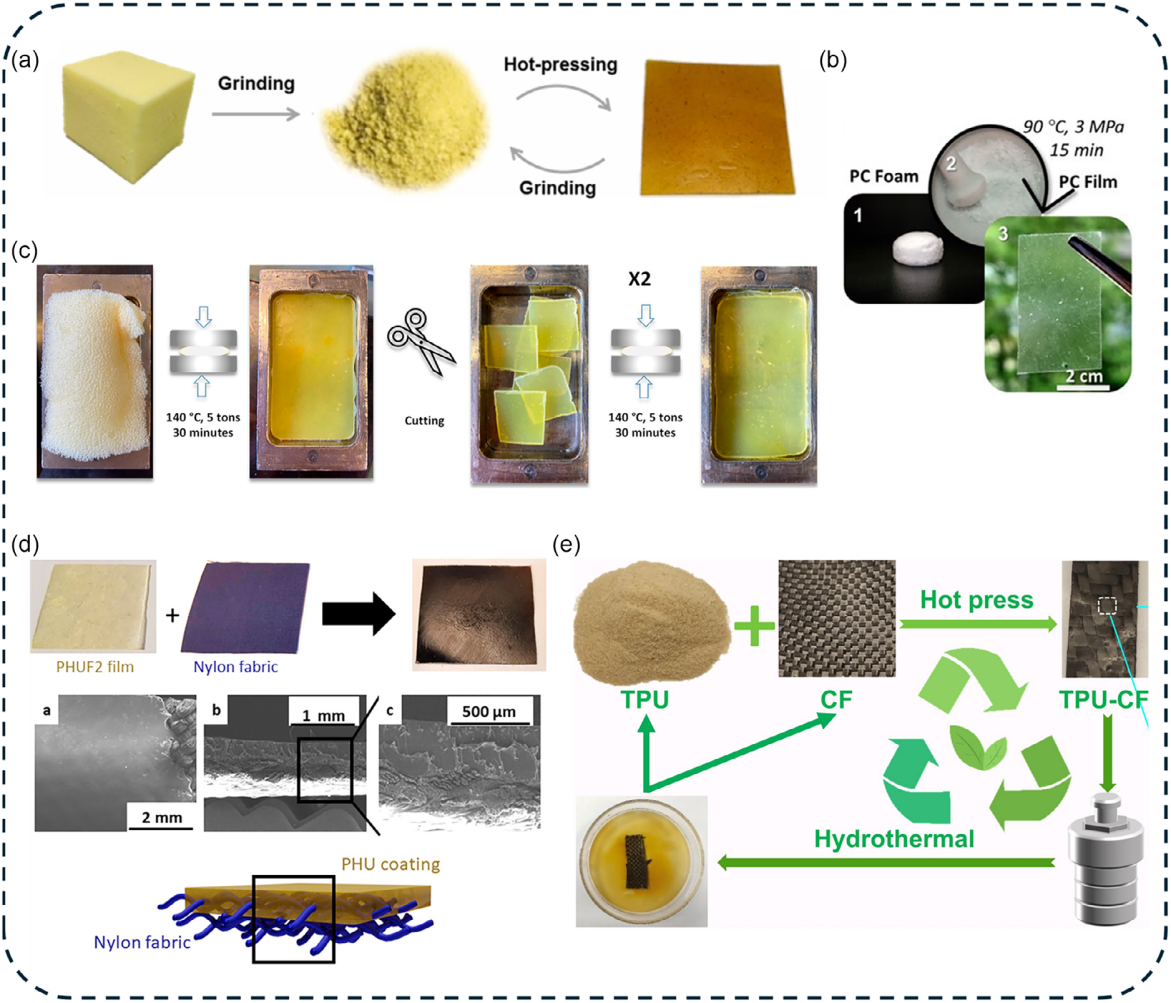
Examples of foam‐to‐film reprocessing of dynamic covalent polymeric foams. (a) Schematic diagram of reprocessing of imine‐based PUF. Reproduced with permission [60]. Copyright 2024, Elsevier. (b) Demonstration of the transcarbonation‐based polycarbonate foam reprocessability by hot pressing. Reproduced with permission [85]. Copyright 2023, Wiley‐VCH. (c) Reprocessing of acetoacetyl‐formed amides‐based PUF into an elastomer, followed by the reprocessing of the obtained elastomer twice. Reproduced with permission [89]. Copyright 2023, American Chemical Society. (d) Fabrication of the nylon/PHU coating composite from recycled NIPUF. Reproduced with permission [52]. Copyright 2022, American Chemical Society. (e) Schematic diagram for preparing carbon fiber/PU composites from recycled PUF. Reproduced with permission [48]. Copyright 2023, Elsevier.

The mechanical recycling of polymer foams via thermocompression represents a time‐efficient and cost‐effective valorization strategy to convert DCPFs into dense polymers while maintaining their intrinsic performance [30, 31, 32, 33, 34, 35, 38, 39, 48, 49, 50, 52, 53, 54, 55, 56, 58, 59, 60, 68, 69, 71, 72, 73, 75, 76, 81, 85, 86, 89, 90, 91]. This method is compatible with standard industrial equipment and typically proceeds under solvent‐ and reagent‐free conditions. While DCPFs containing active DCBs can be directly reprocessed from foam scraps, those containing latent DCBs require addition of external catalysts. Prior to thermocompression, foam grinding is needed to remove trapped air and facilitate mold packing. This step also enhances interfacial bond exchange reactions during reprocessing. Optimal reprocessing conditions were defined by applying pressure (0.03–30 MPa) and selecting a reworking temperature window that satisfies three criteria: (1) above the *T*_g_ of the network, (2) within the active range of the dynamic exchange, and (3) below the onset of thermal degradation. Under these conditions, dynamic exchange reactions redistribute internal stress and promote network rearrangement at the interfaces between cell‐walls, resulting in homogeneous and self‐standing films with well‐defined geometry (Figure 7a–c). Systems with fast dynamic chemistries can achieve complete reprocessing within minutes [73], whereas slower systems may require several hours to reach full densification. Ideally, reprocessed materials retain their cross‐link density and thermo‐mechanical properties after multiple recycling cycles. However, irreversible side reactions at high temperature or during prolonged processing times may alter the network topology, leading to reduced performances [38]. Mechanical recycling of DCPFs can also be achieved with advanced processing technologies such as twin‐screw extrusion [36, 37] or pseudo‐injection molding [73], ensuring suitable reintegration into manufacturing processes. Foams can be successfully reprocessed into films for textile laminates (Figure 7d) [52, 72] or carbon–fiber‐reinforced composites (Figure 7e) [48].

Recycling processes inevitably affect the properties of DCPFs as repeated network rearrangements, thermal exposure, and processing conditions can alter their structure and performance. While the efficiency of the recycling process can be readily evaluated by comparing the recycled resin to its neat counterpart, identifying a suitable point of comparison becomes more challenging when a foam is reprocessed into a film. This difficulty arises because the mechanical properties of foams and films are fundamentally different and therefore not directly comparable. To bridge this gap, the Gibson–Ashby model provides a useful framework for relating the properties of foams to those of their corresponding dense materials [93]. This model establishes scaling‐law relationships that links the macroscopic properties of foams to those of the corresponding dense materials. It is widely used to predict the mechanical performance of polymer foams and to guide their design toward tunable and application‐specific properties. The Gibson–Ashby relationship expresses the mechanical properties of the foam and the bulk material as a function of the relative density, as shown in Equation (1):

(1) $$\frac{P_{\text{foam}}}{P_{\text{solid}}}\propto c{\left(\frac{\rho_{\text{foam}}}{\rho_{\text{solid}}}\right)}^{n}$$

where “*P*” represents a mechanical property evaluated under linear elastic deformation (e.g. modulus or strength), “*ρ*” the relative density, “c” a constant depending on cell geometry (*c* ≈  1 for open‐cell foams), and “*n*” the exponent describing the deformation mode of the bulk material. Torkelson and coworkers applied the cubic model for open‐cell cellular materials to tailor and predict the properties of NIPU‐based DCPFs [53]. They used this model into two experimental systems to either: (1) tailor NIPU‐foams with a targeted storage modulus (*E*′_target_ = 0.2 MPa at 80°C) or (2) to predict a target density range (*ρ*_target_ = 0.37–0.40 g·cm^−3^). In both cases, minimal deviation between predicted and experimental values was observed (*E*′_experimental_ = 0.22 MPa at 80°C; *ρ*_experimental_ = 0.37 g·cm^−3^), evidencing that the Gibson–Ashby model is thus not only a predictive tool but also a powerful design framework. It provides a quantitative basis for engineering recycled foams that do not merely match the original material properties, but can be strategically optimized to deliver enhanced or customized functionality.

#### 4.3.2 Foam‐to‐Foam Recycling

**FIGURE 8:**
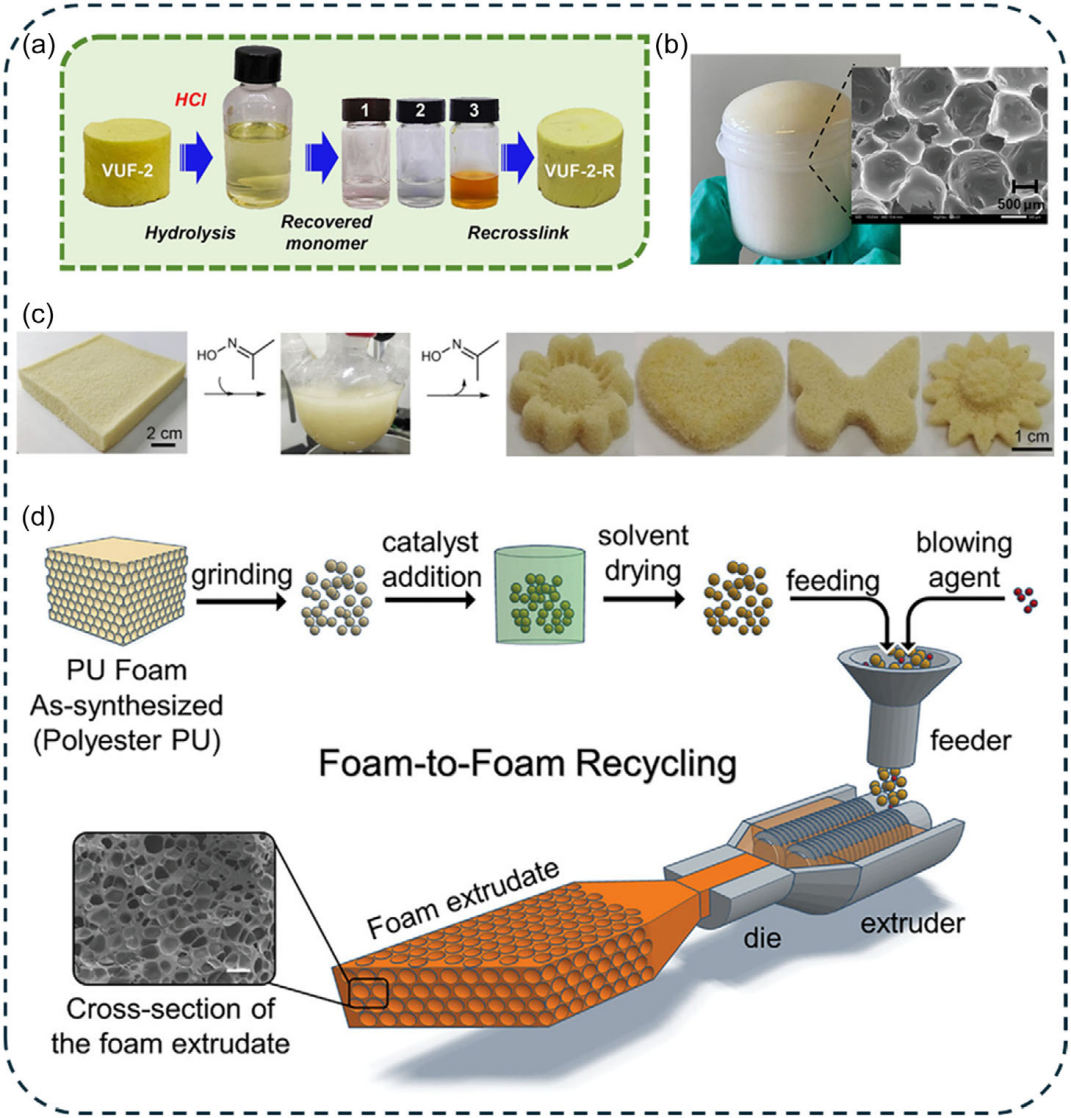
Examples of foam‐to‐foam recycling of dynamic covalent polymeric foams. (a) Closed‐loop recovery of vinylogous urethane‐based foam. Reproduced with permission [88]. Copyright 2024, Elsevier. (b) Image of regenerated *β*‐aminoester‐based PUF and an SEM image in secondary electron detection mode. Reproduced with permission [91]. Copyright 2024, Wiley‐VCH. (c) Photographs of the original PUF, the acetoxime/particle mixture after deconstruction, and the regenerated PUFs. Reproduced with permission [92]. Copyright 2025, Springer Nature. (d) Schematics of the foam‐to‐foam recycling process of urethane exchange‐based PUF using a twin‐screw extruder. Reproduced with permission [41]. Copyright 2023, Wiley‐VCH.

DCBs also bring innovative solutions to regenerate thermosetting foams from their own wastes, representing a true paradigm shift [88, 91, 92]. This approach requires breaking the foam scraps at the molecular level, enabling their reuse either on their own or in combination with fresh reactants. An efficient strategy that has been reported is the solvent‐assisted depolymerization which intends to fragment polymer networks into lower‐molecular‐weight species of reduced viscosity. Several solvent‐mediated depolymerization routes have been developed, including acidolysis, alcoholysis, or hydrolysis, to cite but a few [2, 6, 94]. However, these processes often require harsh conditions that hinder the complete recovery of precursors or compromise product quality. A more selective strategy takes advantage of the reversible nature of dynamic cross‐links. Hydrolysis of linkages such as ester [67], carbonate [85], vinylogous urethane [88], or imine [77] have been shown to effectively deconstruct foams into smaller fragments. Alternatively, depolymerization can be triggered by disrupting the stoichiometric balance between exchange partners through the introduction of small‐molecules capable of reversible bond exchange. Examples include carbamates for carbamate exchange [95], alcohols for transesterification [66, 91] or transcarbamoylation [91], and amines for imine exchange [76, 77]. The functional monomers and oligomers recovered through these processes can be reused, either alone [88] or in combination with fresh precursors [91], to re‐build polymer networks and regenerate foams (Figure 8a,b).

Despite encouraging results, large‐scale implementation is limited by the need for extensive purification steps or considerable amounts of fresh monomers and catalysts. An elegant approach was reported by Xie et al. to tackle these issues [92]. They started from PUF wastes that they chemically degraded using acetoxime to shift the urethane dynamic equilibrium toward network dissociation. The release of acetoxime during foam regeneration from recovered scraps acts as a blowing agent and shifts the dynamic equilibrium toward network re‐association (Figure 8c). This zero‐waste route enables the regeneration of polymer foams with properties comparable and even superior to those of the original materials, marking an important step toward true circularity in polymer foams.

Nondestructive recycling strategies have also been explored to enable refoaming of DCPFs [41, 73]. Ditchel et al. exploited the heat‐induced flowability of PU dynamic networks to regenerate PUFs through continuous twin‐screw extrusion (Figure 8d) [41]. Ground foam wastes were first combined with a zirconium‐based catalyst to accelerate the kinetic of carbamate exchange, followed by addition of azodicarbonamide chemical blowing agent and continuous extrusion. Within the temperature range that enables both carbamate exchange and blowing‐agent vaporization, dynamic bond exchange promoted extensional flow, enabling cell growth and the formation of regenerated foams with morphology and mechanical properties comparable to the original. This method, also compatible with foam injection molding, was also effective for foams that intrinsically contain DCBs [73]. On‐going optimization could further extend this innovative approach to other classes of polymer foams and facilitate its implementation at the industrial scale.

## 5 SWOT Analysis of Dynamic Covalent Polymeric Foams

**FIGURE 9:**
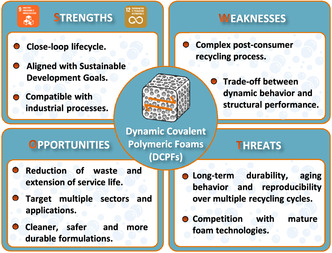
SWOT analysis of dynamic covalent polymeric foams.

DCPFs are attracting growing interest due to their potential to enable recyclable, reconfigurable, and lightweight materials. However, their development remains at an early stage, and their long‐term relevance extends beyond intrinsic chemical performance to encompass broader considerations such as sustainability, scalability, and industrial feasibility. In this context, applying a SWOT analysis provides a structured and comprehensive framework to critically assess the current status of DCPFs, identifying pros and cons, and providing an objective comparison to conventional polymeric foams. This approach offers a clear overview of their inherent advantages, identifies the key scientific and technological limitations that must be addressed, and highlights external factors that may either accelerate or hinder their wider adoption (Figure 9) [24].

### 5.1 Strengths

A clear strength of DCPFs lies in their intrinsic recyclability and capacity for closed‐loop material lifecycle. In fact, solutions for recycling polymer foams already exist such as melt‐processable thermoplastic foams and chemically recyclable thermosets. However, if thermoplastic foams often suffer from limited thermal resistance and mechanical performance under demanding service conditions, the breakdown of crosslinked thermoset networks typically requires energy‐intensive processes that promote irreversible depolymerization. In contrast, DCPFs retain the intrinsic advantages of crosslinked thermosets such as superior thermal stability, dimensional integrity, and creep resistance, while enabling multiple reprocessing and repair cycles through bond‐exchange reactions. It preserve network integrity, particularly in associative exchange reactions. As such, DCPFs represent a cost‐effective trade‐off between circularity efficiency and long‐term material performance. DCBs enable foams to be reprocessed into new materials that preserve or even enhance the properties of their parent networks. This behavior significantly reduces material replacement and waste generation, thereby mitigating environmental impact and supporting the principles of the circular polymer economy. Unlike conventional thermosetting foams, which are typically crosslinked irreversibly and therefore landfilled after use, DCPFs can undergo controlled bond exchange reactions, allowing reshaping, healing, and reconfiguration under relatively mild thermal or chemical stimuli. Harnessing dynamic exchange reactions across multiple stages of the foam lifecycle also unlocks new functional capabilities in cellular materials. These reactions can play an active role not only in recycling but also during foam formation, stabilization, and post‐processing. It allows porosity' fine‐tuning and the opportunity to customize the topology of the foams. Another strong advantage lies in the compatibility of DCPFs with existing industrial practices. The foams’ precursors can be seamlessly integrated into formulations without disrupting established production methods. This formulation' flexibility facilitates the transition toward low‐carbon manufacturing.

### 5.2 Weaknesses

Despite these promising features, several key challenges remain. One major limitation lies in the complexity of the post‐consumer recycling processes. While thermo‐compression offers a relatively simple route for foam‐to‐film conversion, true foam‐to‐foam or closed‐loop recycling often necessitates solvent‐assisted depolymerization and isolation steps. Moreover, residual catalysts or small‐molecule exchange agents may introduce additional purification requirements or regulatory constraints, particularly for applications in healthcare, packaging, or consumer goods. Another fundamental weakness arises from the intrinsic trade‐off between dynamic behavior and structural performance. Increasing the density of exchangeable bonds enhances reprocessability and self‐healing capabilities, but it can compromise mechanical robustness, dimensional stability, and creep resistance under operational conditions. For example, depolymerization in dissociative DCPFs induces thermoplastic‐like flow behavior at elevated temperatures. At the macroscopic scale, this results in a loss of dimensional stability and the collapse of the polymer foams. To prevent this deconstruction, most of DCPFs rely on associative exchanges, or on combined dissociative and associative pathways. Indeed, many dynamic covalent bonds can undergo either associative or dissociative exchanges. However, in most cases, the associative exchange pathway is dominant, thereby preserving network integrity during processing and across multiple recycling cycles. Conversely, restricting bond dynamics may preserve structural integrity but reduces recyclability and adaptability. This balance becomes even more critical in applications requiring long‐term durability or exposure to fluctuating thermal and environmental conditions. Evaluating recyclability by comparing the properties of the original and reprocessed materials over successive recycling cycles represents an effective approach to assess the recovery of crosslink density and the retention of the foams’ performance. These weaknesses collectively underscore the need for further advancements in molecular design, processing strategies, and evaluation standards to fully unlock the potential of DCPFs in sustainable and high‐performance applications.

### 5.3 Opportunities

DCPFs are exceptionally well positioned to benefit from the growing demand for circular and nontoxic materials driven by regulatory and societal demands. The current foam market is dominated by polyurethane which depends on hazardous chemicals such as isocyanates and cannot be structurally recycled. These materials are increasingly restricted under frameworks such as the REACH regulation and the Green Deal. Advantageously, DCPFs benefit from compatibility with safer non‐isocyanate PU alternatives such as the thiol‐induced ring‐opening of cyclic carbonate, which, in addition to address the toxicity and sustainability concerns associated with isocyanates, also address issues associated with limited end‐of‐life options of PUFs. In this context, DCPFs offer a direct response to regulatory and market needs, enabling lightweight materials that can be repaired, reprocessed, and recycled without the use or the release of harmful substances. A major emerging opportunity lies in the development of internally catalyzed dynamic covalent chemistries, where catalytic functions are embedded within the polymer’ backbone or activated through neighboring group participation [21, 22]. This eliminates the need for external catalysts, many of which are toxic, poorly compatible with industrial processes, or known to cause long‐term degradation in polymer networks. Internally catalyzed systems therefore open the way to cleaner, safer, and more durable formulations, aligning DCPFs with future requirements for low‐toxicity and fully circular materials. Beyond regulatory compliance, DCPFs align with broader sustainability targets such as the United Nations Sustainable Development Goals (SDGs) and the transition to a circular polymer economy. Their compatibility with bio‐based monomers or renewable additives positions them as attractive materials for low‐carbon manufacturing pathways. At the same time, their ability to reconfigure microstructure through dynamic exchange reactions enables adaptive design strategies that can reduce manufacturing waste and extend service life. The dynamic nature of these materials also enables new functionality across multiple sectors where lifespan, adaptability, and material recovery are critical. Reprocessed DCPFs have already demonstrated utility in textile manufacturing [52, 72] and advanced composites [48], while their inherent recyclability is inspiring innovative designs for solar energy systems [75] and advanced functional materials [57, 66, 76]. By engineering foams that can be repaired or reconfigured on‐demand, DCPFs unlock recyclability in novel applications such as adaptative insulation technologies (e.g. electromagnetic interference shielding composite foam [44, 76]), energy storage (e.g. solar panel attachment [68]), soft robotics (e.g. piezoresistive auxetic composite foam [57]), and wearable systems (e.g. ferromagnetic syntactic foam [66]). Furthermore, their compatibility with digital manufacturing techniques such as additive foaming or 3D printing facilitates the development of lightweight and multifunctional components tailored for specific performance profiles.

### 5.4 Threats

DCPFs are still at an early stage of technological development, with most systems demonstrated only at the laboratory or pilot scale. At this low technology readiness level (TRL), key uncertainties remain regarding their long‐term durability, aging behavior, and reproducibility over multiple recycling cycles. Such data are essential to gain trust from end‐users, particularly in sectors like transportation, construction, and aerospace, where performance reliability and safety certification are mandatory. Due to their low TRL development, DCPFs face significant competition from mature foam technologies such as polyurethane systems. These conventional materials benefit from decades of optimization, deeply established manufacturing infrastructure, and global supply chains that ensure low cost and large‐volume production. Even if DCPFs demonstrate superior sustainability, their adoption may be hindered by the capital investment required to adapt existing processing lines. Additionally, non‐isocyanate polyurethanes, melt reprocessable thermoplastics, or improved chemically recyclable thermosets are more developed sustainable technologies that could capture the sustainability‐driven market demand before DCPFs reach industrial maturity. Comprehensive life‐cycle assessments and cost–benefit analyses are urgently needed to position DCPFs as credible alternatives to current systems. Unless DCPFs can deliver demonstrable performance consistency across both virgin and recycled states, their large‐scale implementation remains a challenge, potentially preventing them from crossing the valley of death.

## 6 Conclusion

DCPFs have the potential to address one of the most persistent challenges in cellular materials science: reconciling lightness, performance, circularity, and sustainability. The reversible nature of their networks enables true material circularity, ensuring that polymer value is preserved across successive use and recovery stages. The development of DCPFs aligns with international sustainability agendas, including the United Nations Sustainable Development Goals, notably Goal 9 (*Industry*, *Innovation*, *and Infrastructure*) and Goal 12 (*Responsible Consumption and Production*). Altogether, the dynamic exchange reactions at the core of these materials establish DCPFs as a promising class of next‐generation cellular materials that unites lightness with circularity. Among the various strategies applied to DCPFs, associative chemistries remain the most widely used, as they allow the dimensional stability of the foams to be maintained over a wide temperature range. Each chemistry has its advantages and limitations, and the choice largely depends on the specifications of the targeted application. Key considerations include whether the foam will be exposed to high temperatures or humidity, or whether the foaming process requires low‐temperature conditions or can tolerate higher processing temperatures. In this context, the emergence of self‐foaming systems, in which the chemical process is triggered by dynamic exchanges, opens new opportunities. Advanced end‐of‐life strategies have been considered, including repairing, rejoining foam scraps, foam‐to‐film reprocessing, or foam‐to‐foam recycling. All these approaches illustrate that clear opportunities exist; however, they still need to be validated at the industrial level, particularly with respect to the suitability of the proposed solutions and processes in terms of energy consumption and the generation of waste or byproducts. The field of DCPFs is still emerging, and it is clear that many challenges remain to be addressed. Future research could focus on broadening the range of compatible catalysts. The most important would be the development of catalysts able to activate dormant DCBs in existing post‐consumer polymer foams. In parallel, recent advances in alternative dynamic exchange chemistries should be explored to optimize the trade‐off between dynamic network rearrangement and long‐term thermal and mechanical stability. Finally, addressing long‐term durability under service conditions and documenting life‐cycle impacts will be critical to support the scalable and sustainable implementation of DCPFs.

## Author Contributions

**Antoine Adjaoud**: writing – original draft (lead). **Anaë Girault‐Fodil**: writing – original draft (equal). **Farida Baraka**: writing – original draft (equal). **Pierre Verge**: funding acquisition (lead), supervision (lead), and writing – review and editing (lead).

## Funding

This work was supported by Fonds National de la Recherche Luxembourg (C24/MS/18975310).

## Conflicts of Interest

The authors declare no conflicts of interests.
